# Estimation of the Intracranial Volume Is Crucial in Multi‐Site Studies: Reliability for Longitudinal Investigations and Traveling Subjects

**DOI:** 10.1002/hbm.70405

**Published:** 2025-11-05

**Authors:** Shinsuke Koike, Norihide Maikusa, Lin Cai, Issei Ueda, Shuhei Shibukawa, Toshihiko Aso, Saori C. Tanaka, Takuya Hayashi, the Japanese Strategic Research Program for the Promotion of Brain Science (SRPBS) DecNef Study Project Group, Brain/MINDS Beyond Human Brain MRI (BMB‐HBM) Study Project Group

**Affiliations:** ^1^ Center for Evolutionary Cognitive Sciences, Graduate School of Art and Sciences The University of Tokyo Tokyo Japan; ^2^ Institute for Diversity & Adaptation of Human Mind (UTIDAHM) University of Tokyo Tokyo Japan; ^3^ The International Research Center for Neurointelligence (WPI‐IRCN) The University of Tokyo, Institutes for Advanced Study (UTIAS) Tokyo Japan; ^4^ Division of Information Science Nara Institute of Science and Technology Nara Japan; ^5^ Faculty of Health Science, Department of Radiological Technology Juntendo University Tokyo Japan; ^6^ Laboratory for Brain Connectomics Imaging RIKEN Center for Biosystems Dynamics Research Hyogo Japan; ^7^ Brain Information Communication Research Laboratory Group Advanced Telecommunications Research Institute International Kyoto Japan; ^8^ Department of Brain Connectomics Kyoto University Graduate School of Medicine Kyoto Japan

## Abstract

Accurate estimation of the total intracranial volume (TIV) is essential in brain magnetic resonance imaging (MRI) studies, particularly for multi‐site longitudinal investigations. This study assessed the validity and reliability of segmentation‐based TIV (sbTIV) implemented in FreeSurfer version 7.2 for large‐scale multi‐site MRI data, by comparing it with the widely used estimated TIV (eTIV). We analyzed 6524 structural MRI scans from two multi‐site projects in Japan, consisting of 30 procedures across 21 sites, 13 MRI machine types, 3 vendors, and 4 protocol categories. We tested the intraclass correlation coefficients (ICCs) between eTIV and sbTIV for each procedure and identified procedural factors affecting these ICCs using a general linear model. Machine‐ and protocol‐specific biases were considered by a traveling subject harmonization approach. To specifically examine the reliability and validity of the longitudinal scans, we employed a general linear mixed model (GLMM). Overall agreement between eTIV and sbTIV was good (ICC = 0.78) but varied across procedures (0.62–0.94). The 1.0 mm isotropic protocol showed the highest reliability. Notably, there was poor consistency in participants with eTIV values of 120,000 mm^3^ or smaller (ICC = 0.053). sbTIV demonstrated superior cross‐procedural consistency in adolescent and adult longitudinal scans compared to eTIV. In longitudinal scans, sbTIV showed greater sex difference and sex‐specific increase for adolescents, and greater consistency for adults, compared to eTIV. sbTIV offers more robust and reliable estimation compared to eTIV, particularly for multi‐site longitudinal studies. These findings highlight the need for careful consideration when interpreting previous multi‐site studies using eTIV.

## Graphical abstract

Estimated TIV (eTIV) as standard in FreeSurfer had good reliability compared to segmentation‐based TIV (sbTIV) implemented in FreeSurfer version 7.2; however, there was poor consistency in participants with eTIV values of 120,000 mm^3^ or smaller. sbTIV demonstrated good cross‐procedural consistency in adolescent and adult longitudinal scans.

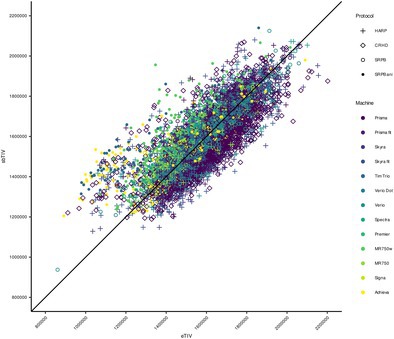

## 1 Introduction

Total intracranial volume (TIV) is associated with body size, such as body height and weight, and is not associated with brain structural and functional characteristics. However, TIV is a key variable for brain MRI studies because some of the structural variables, such as cortical surface area and cortical and subcortical volume, are often adjusted using TIV [Cai et al. 2024; Zhu et al. 2024]. Previous studies have demonstrated strong associations between TIV and these structural measures, with large effect sizes [Barnes et al. 2010; Im et al. 2008]. Moreover, individuals with mental illness have been reported to exhibit smaller TIV compared with healthy controls [ENIGMA Clinical High Risk for Psychosis Working Group et al. 2021; ENIGMA Schizophrenia Working Group et al. 2016; Okada et al. 2016]. Thus, the validity and reliability of the TIV estimation are crucial in a multi‐site study and longitudinal cohort. In particular, TIV estimation is sensitive in adolescent brain development studies because physical development affects TIV increase, but structural features mostly decrease [Bethlehem et al. 2022; Cai et al. 2024; Zhu et al. 2024].

In large‐scale investigations, FreeSurfer [Fischl 2012] is a widely used brain preprocessing software; however, several issues have been identified with the estimated TIV (eTIV) calculated using T1‐weighted images in the software [Buckner et al. 2004]. FreeSurfer employs a registration‐based method which uses indirect scaling through affine transformation to a standard template, resulting in lower TIV estimation accuracy compared to manual segmentation and potentially introducing systematic errors (Figure S1) [Malone et al. 2015; Nerland et al. 2022]. Furthermore, FreeSurfer demonstrated poor longitudinal consistency in eTIV, with theoretically stable adult TIV values varying across different imaging time points [Ma et al. 2019]. Consequently, caution is recommended when using FreeSurfer‐based eTIV, particularly in multi‐site or long‐term neuroimaging research.

Several brain imaging software packages have therefore adopted segmentation‐based methods to estimate TIV. These approaches first segment brain images into tissue compartments and then calculate volumetric estimates of the intracranial cavity. For example, Statistical Parametric Mapping 12 (SPM12, https://www.fil.ion.ucl.ac.uk/spm/) estimates TIV within a unified framework of segmentation and spatial normalization. The volumes estimated by SPM12 have shown higher reliability relative to manually traced volumes, outperforming estimates obtained using SPM8 and the eTIV derived from FreeSurfer [Malone et al. 2015]. Sequence Adaptive Multimodal Segmentation (SAMSEG) implemented based on FreeSurfer provides segmentation‐based total intracranial volume (sbTIV) by calculating direct segmentation within the skull [Cerri et al. 2023; Puonti et al. 2016]. sbTIV relies on structure segmentations including the cerebrospinal fluid and other intracranial non‐brain structures, and can be calculated from T1‐weighted image only or T1‐weighted and T2‐weighted multimodal images using Samseg function implemented from FreeSurfer version 7.2. Previous studies showed that these segmentation‐based TIV estimates were highly correlated; however, a potential limitation is that the estimated volume might be underestimated in individuals with severe atrophy (e.g., dementia) [Nerland et al. 2022].

Here, we aim to test whether sbTIV would demonstrate greater validity and reliability compared to eTIV using multi‐site large‐sample data from the Japanese Strategic Research Program for the Promotion of Brain Science (SRPBS) DecNef [Tanaka et al. 2021] and the Brain/MINDS Beyond Human Brain MRI (BMB‐HBM) study project [Koike et al. 2021], consisting of 6524 structural brain scans using 30 procedures (i.e., machines and protocols). In this study, we also intended to confirm the validity and reliability of the sbTIV in various repeated‐measure datasets scanned using different procedures for traveling subjects (TS), adolescents, and adults [Koike et al. 2021; Koike et al. 2025].

## 2 Methods and Materials

### 2.1 Datasets

**FIGURE 1:**
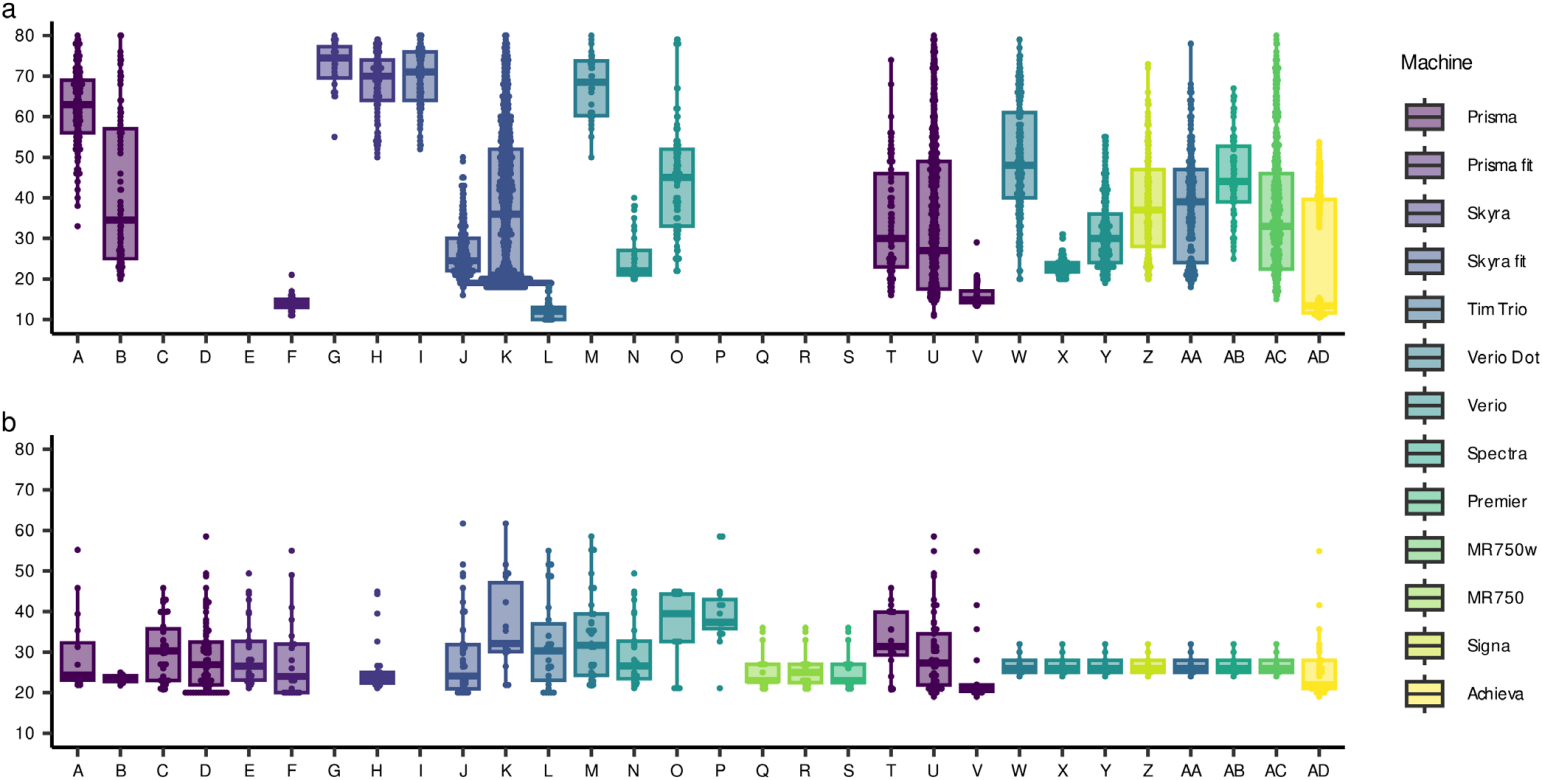
Age distribution by procedure. The age distribution of participants at the time of MRI scanning is shown for the main dataset (a) and the traveling subject (TS) dataset (b). As summarized in Table 1, Procedures A–S, T and U, V–Z, and AA–AD employed the HARP, CRHD, isotropic SRPB, and anisotropic SRPB protocols, respectively.

Ethical approval was obtained from the Ethics Committee of the University of Tokyo (Approval No. 24‐496) and the ethical committees at the measurement sites. All participants provided written informed consent prior to participating in the study. A total of 6641 brain scans from the SRPBS DecNef and BMB‐HBM study projects, 6524 structural brain scans using 30 procedures were used in this study. Of these, 5890 structural brain scans were collected from 4435 participants (mean (SD) age = 32.0 (16.8) years, range 10.5–80.1; female = 1904, 48.8%) using 23 procedures (Table 1 and Figure 1a). In addition, 634 TS scans were used from 134 participants, 28 procedures (age at the initial scan = 29.0 (9.6) years, range 19.0–61.7, female = 49, 36.6%, mean scans = 4.7, SD = 2.0, range 2–9; Figure 1b). To test the effect of body size on TIV estimation, body height (BH) and body weight (BW) were measured on the days of MRI scans in two procedures (Procedure U and V, *n* = 1546, BH: mean ± SD = 165.4 ± 8.8 cm, BW: mean ± SD = 60.1 ± 13.6 kg).

**TABLE 1:** Demographic characteristics and scan procedures.

Procedure	A	B	C	D	E	F	G	H	I	J	K
Site code	JTD	UOS	UTI	UTK	ATR	TMG	KPUM	UKY	NCNP	SWA	UHI
Participants, *n*	179	78	0	0	0	10	34	94	98	209	702
Female, %	94, 52.5	42, 53.8	NA	NA	NA	6, 60.0	17, 50.0	53, 56.4	46, 46.9	52, 24.9	361, 51.4
Age, mean ± SD	63.2 ± 9.4	41.0 ± 19.1	NA	NA	NA	13.1 ± 2.2	74.4 ± 6.5	68.8 ± 8.9	71.0 ± 8.1	26.4 ± 6.3	35.1 ± 17.0
Scans, *n*	192	83	0	0	0	29	34	106	98	248	976
TS scans, *n*	16	13	39	79	32	21	0	25	0	42	18
Data source	BMB										
MRI vendor	Siemens										
Model	Prisma				Prisma fit		Skyra		Skyra fit		
Matrix	320 × 300										
Voxel size (FH × AP × RL), mm^3^	0.8 × 0.8 × 0.8										
Repetition time, ms	2500										
Echo time, ms	2.18										
Flip angle, °	8										

Procedure	L	M	N	O	P	Q	R	S	T	U
Site code	TMG	NCNP	ATR	KRC	UNG	CHB	KEO	UTH	UTI	UTK
Participants, *n*	49	31	34	40	0	0	0	0	87	788
Female, %	27, 55.1	11, 35.5	15, 44.1	21, 52.5	NA	NA	NA	NA	50, 57.5	358, 45.4
Age, mean ± SD	12.3 ± 2.3	67.9 ± 8.6	25.4 ± 6.1	44.6 ± 13.3	NA	NA	NA	NA	33.9 ± 13.9	43.1 ± 16.3
Scans, *n*	49	31	34	107	0	0	0	0	89	1613
TS scans, *n*	30	32	31	15	15	16	15	15	20	49
Data source	BMB									
MRI vendor	Siemens				GE				Siemens	
Model	Tim Trio	Verio Dot	Verio		MR750			Premier	Prisma	
Matrix	320 × 300				320 × 320				320 × 300	
Voxel size (FH × AP × RL), mm^3^	0.8 × 0.8 × 0.8				0.8 × 0.8 × 0.8				0.8 × 0.8 × 0.8	
Repetition time, ms	2500				2512		2828	2487	2400	
Echo time, ms	2.18				2.36		6.06	3.40	2.22	
Flip angle, °	8				8				8	

Procedure	V	W	X	Y	Z	AA	AB	AC	AD
Site code	UTK	COI	ATR	SWA	HUH	UKY	HKH	UTH	YAC
Participants, *n*	144	186	100	232	120	217	62	419	522
Female, %	66, 45.8	114, 61.3	21, 21.0	34, 14.7	60, 50.0	96, 44.2	30, 48.4	203, 48.4	359, 68.8
Age, mean ± SD	15.4 ± 2.1	49.8 ± 13.6	23.0 ± 2.3	31.4 ± 8.8	38.5 ± 12.8	38.1 ± 13.1	45.1 ± 10.5	36.9 ± 15.8	24.9 ± 15.7
Scans, *n*	166	186	100	232	120	217	62	425	693
TS scans, *n*	19	9	9	9	9	9	9	9	29
Data source	BMB	SRPBS							BMB, SRPBS
MRI vendor	Siemens			GE		Siemens		GE	Philips
Model	Prisma	Verio Dot	Verio	Signa HDxt		Tim Trio	Spectra	MR750w	Achieva
Matrix	256 × 256	240 × 256	256 × 256			240 × 256	320 × 320	256 × 256	
Voxel size (FH × AP × RL), mm^3^	1.0 × 1.0 × 1.0					1.0 × 0.9375 × 0.9375	0.8 × 0.75 × 0.75	1.0 × 1.0 × 1.2	
Repetition time, ms	1900	2300	2250	2300	6.8	2000	1900	7.7	6.8
Echo time, ms	2.53	2.98	3.06	2.98	1.93	3.4	2.38	3.1	3.1
Flip angle, °	9	9	9	9	20	8	10	11	9

*Note:* For Procedure A to U, T2 weighted and field map correction images were also obtained in the following parameters; T2 weighted images—matrix: 320 × 300 (Procedure Q, R, and S: 320 × 320), voxel size: 0.8 × 0.8 × 0.8 mm, repetition time: 3200 ms (Procedure Q, R, and S: 3294–3296 ms), echo time: 562–565 ms (Procedure Q, R, and S: 59.5–61.7 ms); field map images—matrix: 86 × 86 (Procedure T and U: 104 × 104), voxel size: 2.4 × 2.4 × 2.4 mm (2.0 × 2.0 × 2.0 mm), repetition time: 6100 ms (8000 ms), echo time: 60 ms (66 ms), flip angle: 90°/180°.

The 30 procedures consisted of 21 sites, 13 machine types, and 3 vendors. The protocols were categorized into four types: Harmonized Protocol (HARP), Connectome Related to Human Disease (CRHD), and isotropic and anisotropic SRPB protocols. The HARP protocol was established in the BMB‐HBM study project [Koike et al. 2021], and 19 procedures, 9 machine types, and 2 vendors were used in this study. The CRHD protocol for Siemens Prisma machine was based on that used in the Human Connectome Project (HCP) [Harms et al. 2018; Somerville et al. 2018], and two sites were used. The SRPBS DecNef project determined standardized protocols [Tanaka et al. 2021]; however, owing to the restriction of clinical sites and the need for continuity from previous projects, there were some variations in parameters across sites. Therefore, we categorized the isotropic SRPB protocol, which has an isotropic 1.0 mm voxel size and consists of five procedures, four machine types, and two vendors, and an anisotropic SRPB protocol consisting of four procedures, four machine types, and three vendors.

### 2.2 Image Preprocessing

Image preprocessing was performed using PreFreeSurferPipeline.sh and FreeSurferPipeline.sh of the HCP pipeline (version 4.3.0) [Glasser et al. 2013] with the standard HCP style for subjects who underwent 3D T1‐ and T2‐weighted images (Appendix S1). The legacy mode pipeline, which includes histogram‐based bias field correction, was applied only to T1‐weighted images. The HCP pipeline used FreeSurfer version 6.0, and eTIV was calculated in FreeSurferPipeline.sh. Additionally, sbTIV was calculated using the ‘run_samseg’ command implemented in FreeSurfer version 7.2 [Cerri et al. 2023; Puonti et al. 2016]. Quality control was manually performed for all preprocessed images by two or more examiners using the ENIGMA QC script (https://enigma.ini.usc.edu/). Because detailed assessments of image quality, boundary segmentation, and neuroanatomical variation were not comprehensively performed for all images in the BMB‐HBM project [Koike et al. 2021], we carried out a brief inspection to verify whether image preprocessing and cortical and subcortical segmentation had been successfully completed. Images that failed to complete preprocessing and/or exhibited substantial errors in boundary segmentation were excluded from further analysis (*n* = 107, 1.6%). To compare the differences between the preprocessing methods, both preprocessing pipelines were used for the TS dataset in Procedures T and U (*n* = 37). In addition, to examine the effects of longitudinal preprocessing pipelines implemented in FreeSurfer and SAMSEG, we performed supplementary preprocessing on six TS images from one participant and four longitudinal images from an adolescent participant scanned over an 8‐year period (Appendix S1).

### 2.3 TS Harmonization

To diminish machine‐ and protocol‐specific measurement bias, TS harmonization was performed for eTIV and sbTIV using procedure‐specific difference [Koike et al. 2021; Koike et al. 2025; Maikusa et al. 2021]. Procedure‐specific differences were estimated using a general linear mixed model (GLMM), including the procedure as a fixed effect and participant as a random effect. We calculated the harmonized eTIV and sbTIV by using the fixed effect of the procedure in the GLMM.

### 2.4 Statistical Analysis

To see the consistency of TIV estimations between the two methods, we tested an intraclass correlation coefficient (ICC) for all samples and each procedure using the ‘irr’ package version 0.84.1 in R version 4.4.2. Significance was set at 0.05, and reliability was evaluated using the following criteria: greater than 0.9, excellent; 0.75–0.9, good; 0.5–0.75, moderate; and less than 0.5, poor. We then tested which procedural factors (i.e., protocol and machine) would affect the ICCs using a general linear model (GLM).

Second, the deviation and absolute error of eTIV from sbTIV as the reference were calculated, and the predictive factors (i.e., protocol, machine, age, and sex) were tested using a GLMM, including procedure and participant as random effects.

Third, whether preprocessing methods would affect deviation and absolute error was tested using repeated‐measures analysis of variance (rmANOVA), including preprocessing methods and TIV estimation methods as within‐subject variables.

Fourth, to determine the reliability of the TIV estimation methods across procedures, we tested the ICC of the average agreement. Since the n of TS scans varied across participants, we selected the participants who received three or more TS scans and performed 1000 random samplings to make the datasets of three scans per participant. Using these samples, we tested the ICC(2,k) for both eTIV and sbTIV. After TS harmonization, the same ICC(2,k) test was performed.

Finally, we tested the non‐linear trajectory throughout the lifespan using a generalized additive mixed model (GAMM), including sex as a linear fixed term, age at MRI scan and age by sex interaction as non‐linear fixed terms, and participant as a random intercept. To see the effects of age and body size on eTIV and sbTIV, we tested Pearson correlation on the available dataset (*n* = 1546). Given that brain atrophy may influence TIV estimation, we further applied a GLM, including age and BH at MRI scan as main effects in the participants over 60 years of age (*n* = 97). To specifically examine the reliability and validity of the longitudinal scans for adolescents and adults, we used a GLMM including age at MRI scan for adolescents or gap from age at initial scan for adults, sex, estimation methods (i.e., eTIV and sbTIV), and their interaction terms as fixed effects, and participants as a random effect.

## 3 Results

### 3.1 The Difference in TIV Estimation Methods Between Procedures

**FIGURE 2:**
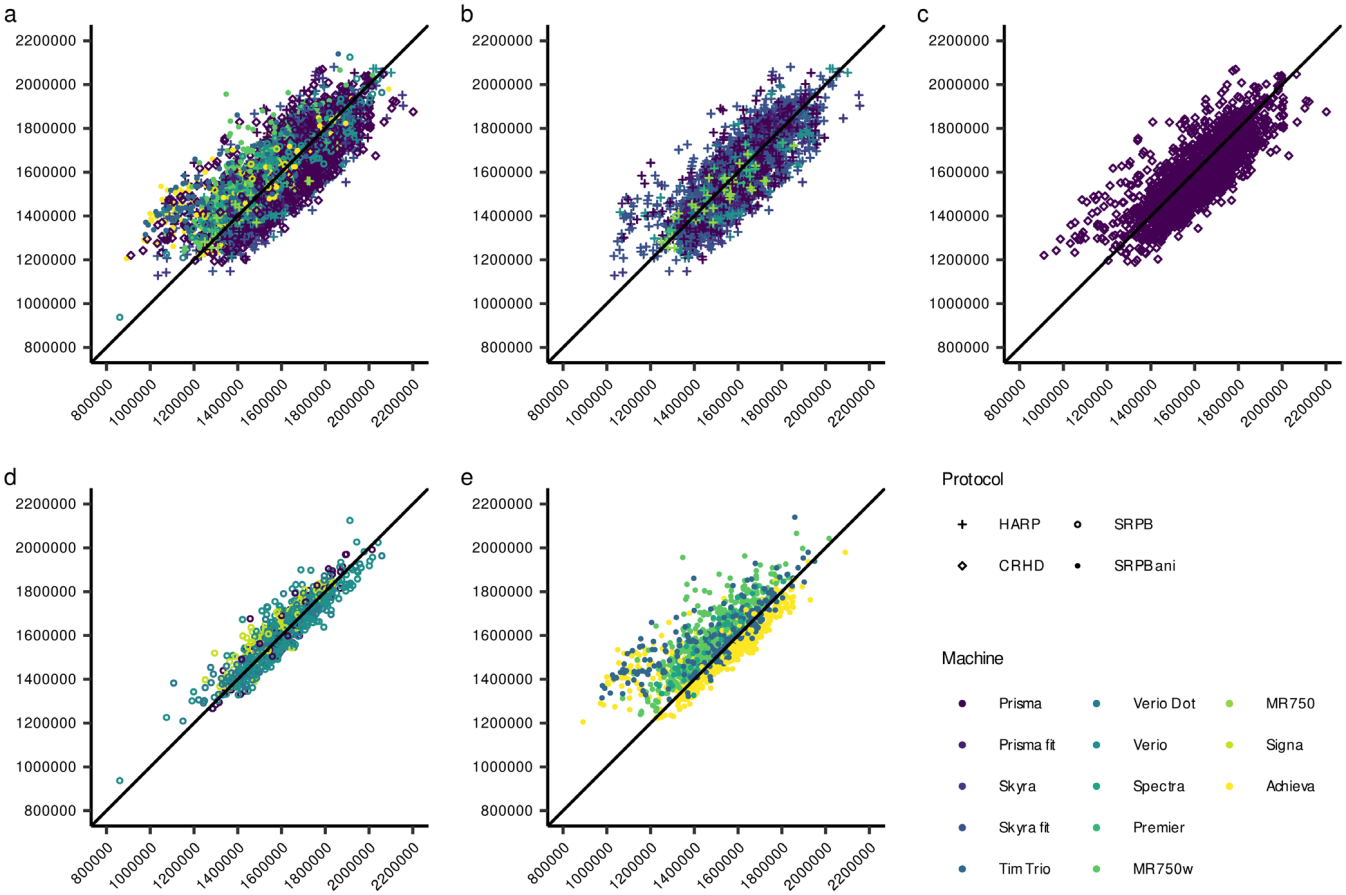
Relationship between eTIV and sbTIV. The relationships were plotted for the overall (a), the HARP (b), CRHD (c), isotropic SRPB (d), and anisotropic SPRB protocols (e). Procedure‐specific relationships are shown in Figure S1.

Overall, the absolute agreement between the eTIV and sbTIV was good (ICC(2,1) = 0.78, *p* < 0.001; Figure 2), but the ICCs varied from good to moderate across procedures (0.62–0.94, all *p* < 0.001; Table 2 and Figure S1). In particular, images with 120,000 mm^3^ or smaller volume of eTIV were under‐estimated and showed no consistency compared to sbTIV (*n* = 165, ICC(2,1) = 0.053, *p* = 0.22, Figure 2). In contrast, the non‐small eTIV subgroup, which had more than 120,000 mm^3^ eTIV volume, also showed various consistencies between sites (*n* = 6357, Overall ICC(2,1) = 0.79, *p* < 0.001; range: 0.67–0.94, all *p* < 0.001). These ICCs showed little improvement compared to those for all samples, except for Procedure AD, which showed a remarkable difference between eTIV and sbTIV for those with a small eTIV (Figure S2).

**TABLE 2:** Absolute agreement, deviation, and absolute error between eTIV and sbTIV.

Procedure	MRI model	Protocol	*n*	ICC(2,1)	ICC(2,1) for non‐small eTIV subgroup	eTIV, mean (SD)	sbTIV, mean (SD)	Deviation, mean (SD)	Absolute error, mean (SD)
Overall			6524	0.78	0.79	1,576,865 (173,438)	1,580,168 (159,199)	−3303 (109,866)	83,434 (71,548)
A	Prisma	HARP	208	0.73	0.75	1,599,380 (162,141)	1,581,799 (158,767)	17,581 (116,702)	94,987 (69,740)
B			96	0.73	0.73	1,549,394 (164,337)	1,555,976 (174,440)	−6582 (125,453)	91,578 (85,483)
C			39	0.82	0.82	1,641,860 (128,342)	1,647,789 (143,599)	−5929 (82,903)	63,079 (53,154)
D			79	0.79	0.80	1,633,306 (167,183)	1,658,247 (175,458)	−24,941 (109,412)	89,861 (66,502)
E	Prisma fit		32	0.77	0.82	1,577,572 (176,220)	1,605,332 (170,213)	−27,760 (116,868)	88,318 (80,012)
F			50	0.84	0.84	1,626,801 (154,359)	1,611,529 (167,832)	15,272 (92,102)	75,890 (53,330)
G	Skyra		34	0.72	0.72	1,616,763 (198,540)	1,513,506 (222,343)	103,257 (132,621)	152,223 (68,554)
H			131	0.78	0.75	1,592,787 (174,094)	1,529,599 (176,943)	63,188 (103,534)	99,121 (69,587)
I	Skyra fit		98	0.72	0.70	1,620,943 (152,804)	1,550,964 (157,049)	69,979 (101,270)	98,687 (73,236)
J			290	0.80	0.80	1,620,575 (162,942)	1,648,909 (175,098)	−28,334 (103,407)	82,662 (68,132)
K			994	0.82	0.82	1,593,172 (170,531)	1,592,131 (162,480)	1041 (101,035)	80,026 (61,632)
L	Tim Trio		79	0.73	0.73	1,568,459 (131,214)	1,519,499 (142,006)	48,960 (91,965)	91,455 (49,135)
M	Verio Dot		63	0.74	0.74	1,660,226 (144,733)	1,595,649 (173,555)	64,577 (102,829)	101,171 (66,413)
N	Verio		65	0.80	0.82	1,595,594 (151,787)	1,562,263 (173,617)	33,331 (98,692)	82,306 (63,153)
O			122	0.83	0.84	1,574,865 (196,048)	1,552,656 (204,050)	22,209 (113,820)	92,755 (69,122)
P			15	0.80	0.80	1,572,121 (135,841)	1,547,831 (152,855)	24,289 (91,294)	77,976 (49,520)
Q	MR750		16	0.93	0.93	1,466,800 (118,347)	1,489,148 (113,967)	−22,348 (40,354)	36,430 (27,305)
R			15	0.82	0.82	1,543,656 (181,127)	1,466,053 (134,828)	77,603 (67,997)	89,691 (49,566)
S	Premier		15	0.89	0.89	1,542,204 (141,395)	1,549,570 (115,785)	−7366 (62,639)	50,756 (34,952)
T	Prisma	CRHD	109	0.79	0.79	1,600,890 (189,146)	1,596,955 (171,844)	3935 (117,098)	92,566 (71,272)
U			1662	0.77	0.77	1,609,330 (169,901)	1,580,947 (157,191)	28,383 (108,742)	90,955 (65,978)
V		SRPB	185	0.94	0.94	1,576,305 (133,584)	1,583,218 (142,064)	−6913 (46,257)	34,676 (31,286)
W	Verio Dot		195	0.92	0.93	1,544,601 (147,316)	1,551,515 (135,287)	−6915 (55,010)	40,857 (37,367)
X			109	0.90	0.90	1,601,287 (142,648)	1,591,055 (131,903)	10,231 (61,200)	45,667 (41,788)
Y	Verio		241	0.91	0.89	1,665,306 (156,320)	1,677,009 (146,352)	−11,703 (64,376)	49,734 (42,404)
Z	Signa HDxt		129	0.87	0.87	1,550,860 (128,219)	1,588,599 (128,343)	−37,739 (56,262)	52,621 (42,545)
AA	Tim Trio	SRPB ani	226	0.62	0.67	1,482,482 (220,790)	1,595,149 (145,683)	−112,667 (135,817)	125,138 (124,368)
AB	Spectra		71	0.78	0.78	1,494,227 (143,588)	1,583,119 (140,009)	−88,893 (54,344)	90,884 (50,894)
AC	MR750w		434	0.66	0.65	1,481,221 (148,512)	1,597,353 (152,177)	−116,131 (81,169)	116,309 (80,913)
AD	Achieva		722	0.74	0.85	1,503,445 (170,190)	1,508,724 (120,239)	−5278 (105,619)	68,589 (80,450)

*Note:* Intraclass correlation coefficients (ICC(2,1)) were also obtained for non‐small eTIV subgroup which includes the samples with more than 1,200,000 mm^3^ of estimated total intracranial volume (eTIV). Deviation was defined as eTIV − sbTIV, and absolute error as |eTIV − sbTIV|.

We performed a GLM using Procedure V, which had the highest ICC across all procedures, as the reference. The GLM showed significantly higher ICCs in the SRPB isotropic protocols compared to the other protocols (Figure 2d), while the other three protocols showed no difference (Table S1). We also found a main effect of the machine, but there seems to be no tendency for the machine vendor or performance. The results were similar when the images were limited to the non‐small eTIV subgroup.

### 3.2 Factors of Deviation and Absolute Error Between TIV Estimation Methods

The mean deviation and mean absolute error also differed across the procedures (Table 2). A GLMM for deviation (i.e., eTIV − sbTIV) as the dependent variable showed that the HARP protocol overestimated eTIV by referring to sbTIV (Table S2). This result was consistent in the non‐small eTIV subgroup (Table S3). In addition, the Skyra machine and female sex overestimated the eTIV (Figure S3).

In terms of absolute error, similar to the findings of the ICC analysis, the SRPB isotropic protocol was the smallest compared to the other three protocols (Tables S4 and S5). Age at MRI scan was positively associated with absolute error (Figure S3). Female sex was positively, but in the non‐small eTIV subgroup, negatively associated with absolute error (*B* = 5496, SE = 2072, *t* = 2.65, *p* = 0.008; *B* = −4846, SE = 1851, *t* = −2.62, *p* = 0.009; respectively).

### 3.3 The Difference Between Preprocessing Methods

An rmANOVA showed a significant preprocessing method × TIV estimation method interaction (*B* = 38,401, SE = 17,979, df = 108.0, *t* = 2.14, *p* = 0.035), but no significant main effects. When the deviation of eTIV as the dependent variable was used, there was a significant main effect of preprocessing method (Intercept: *B* = 5500, SE = 15,010, df = 44.2, *t* = 0.37, *p* = 0.72; Preprocessing method: *B* = −38,401, SE = 9657, df = −3.98, *p* < 0.001). These results suggest that eTIV was under‐estimated when using the legacy mode pipeline (Figure S4).

### 3.4 Reliability of TIV Estimation Methods Across Procedures Using TS Dataset

**FIGURE 3:**
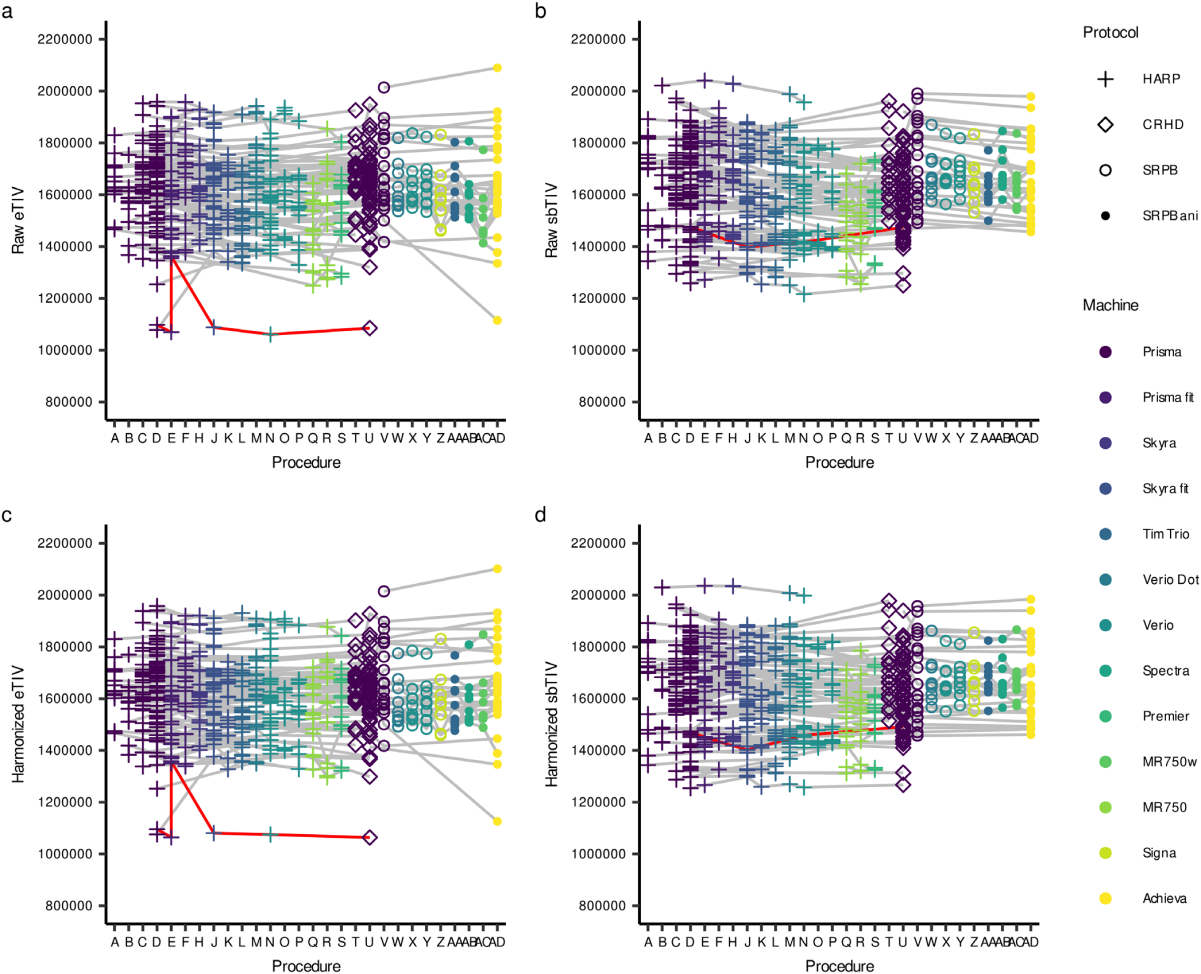
Relationship between eTIV and sbTIV by preprocessing for traveling subject samples. The red line shows an example of an incorrect eTIV estimation across the procedures from one TS participant.

The 1000 random sampling ICC(2,k) showed excellent results for eTIV and sbTIV (mean = 0.969, SD = 0.006; mean = 0.984, SD = 0.001; respectively). However, raw eTIV and sbTIV both included measurement bias, and the eTIV of some TS samples was inconsistent across procedures (Figure 3). After harmonization, the measurement bias successfully diminished, especially in sbTIV, and the ICC(2,k) increased and showed excellent results (eTIV: mean = 0.976, SD = 0.006; sbTIV: mean = 0.989, SD = 0.001).

For the six TS images from one participant shown by the red line in Figure 3 (eTIV = 1,060,642–1,362,655 mm^3^), we applied longitudinal pipelines to calculate both eTIV and sbTIV. The results showed that longitudinal pipeline‐processed eTIV was comparable to sbTIV (1,468,940 mm^3^ vs. 1,398,074–1,475,043 mm^3^) although these eTIV values were identical across different procedures (Figure S5). In contrast, longitudinal pipeline‐processed sbTIV increased compared to standard sbTIV and more approximated harmonized sbTIV. However, a limitation of the longitudinal pipelines was evident in the adolescent longitudinal scans, where a developmental trajectory appears flat for eTIV and attenuated for sbTIV.

### 3.5 eTIV and sbTIV Through the Life Span

**FIGURE 4:**
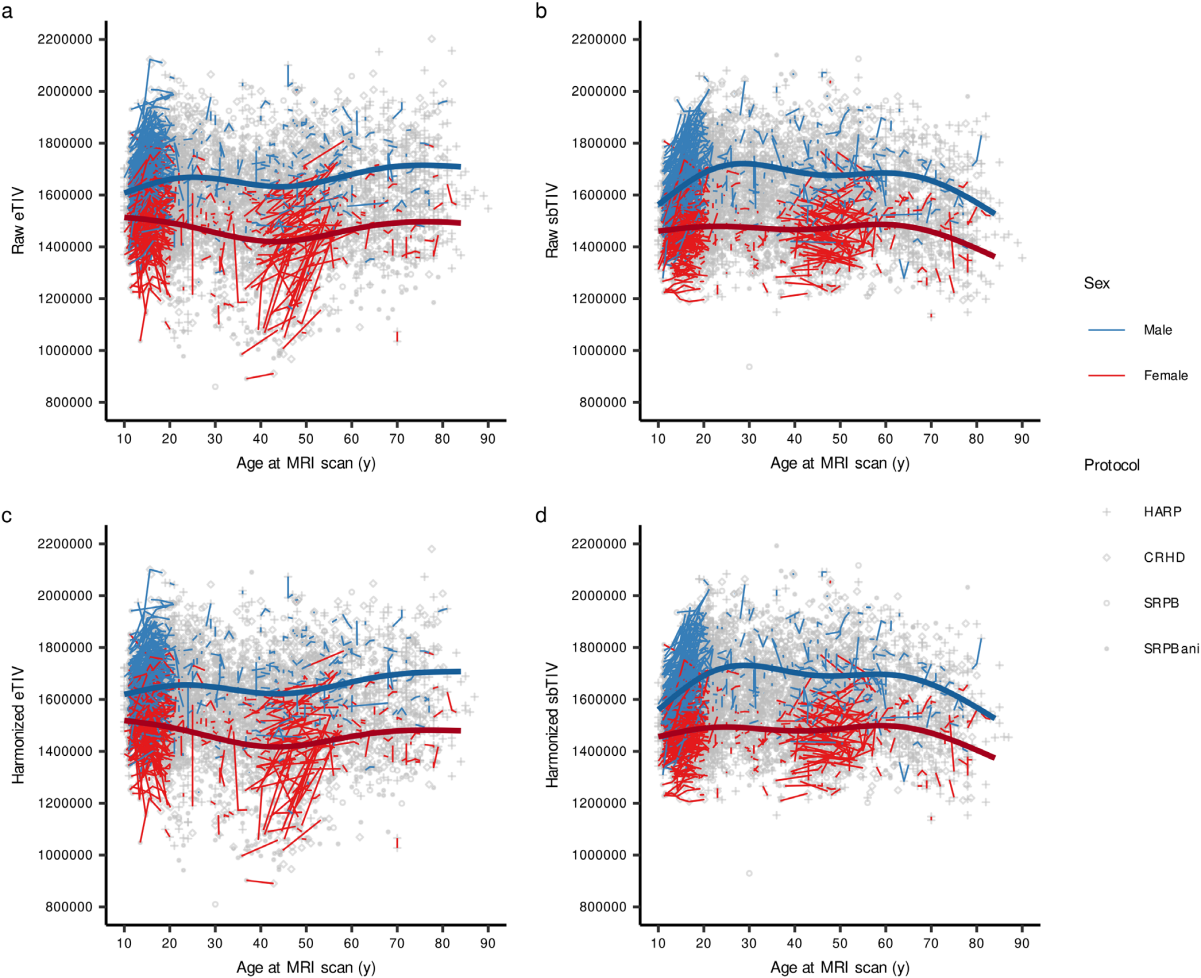
eTIV and sbTIV through the lifespan. The lines show repeated measures from the same participant.

GAMM showed non‐linear trajectories of eTIV and sbTIV before and after TS harmonization throughout the life span (Figure 4). When observing the average trajectory in females, eTIV slightly decreased from age 10 to 40 years, had a dip around age 45, and then increased to age 80, whereas sbTIV changed little to age 70. The reason for the different trajectory patterns is partly seen in the lines of repeated measures, showing a dramatic increase in eTIV of age 40s females. These data mostly came from the initial scans using Procedure AD and the follow‐up scans using Procedure U. These results did not change even when TS harmonization was performed. For males, the increase in sbTIV during adolescence was greater than that in eTIV.

Pearson correlation analyses revealed significant associations of age, BH, and BW with eTIV (*r* = −0.053, 0.45, and 0.33; *p* = 0.038, < 0.001, and < 0.001, respectively), and significant associations of BH and BW with sbTIV (*r* = 0.63 and 0.49; both *p* < 0.001). Among participants over 60 years of age (*n* = 97), GLMs indicated significant main effects of age and BH on eTIV (*B* = 8123, SE = 2965, *t* = 2.74, *p* = 0.007; *B* = 11,629, SE = 2134, *t* = 5.5, *p* < 0.001; Figure S6), whereas for sbTIV only BH showed a significant effect (*B* = 11,163, SE = 354, *t* = 31.5, *p* < 0.001), with no significant effect of age (*p* = 0.49). These findings suggest that sbTIV provides a more robust estimate of intracranial volume in older individuals with relatively smaller brain volumes.

**FIGURE 5:**
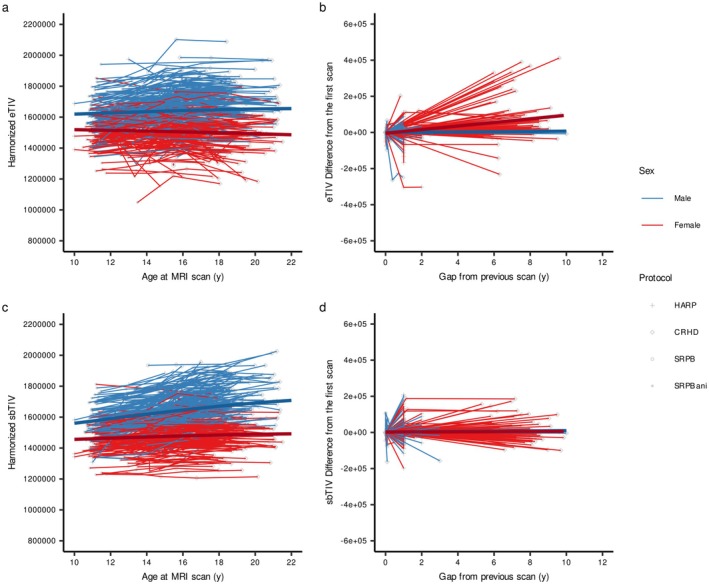
Reliability of the eTIV and sbTIV using repeated‐measure samples. Left panels show adolescent trajectories of eTIV (a) and sbTIV (c) on longitudinal scans. The right panels show the spaghetti plots in eTIV (b) and sbTIV (d) for adult participants with longitudinal scans.

These characteristics were prominent in the longitudinal scans (Figure 5). A GLMM for adolescent longitudinal measurements showed significant main effects of age and estimation method, but not sex, a significant age by sex interaction, and significant interaction terms regarding the estimation method (Table S6). Post hoc models showed that sex difference and sex‐specific increase during adolescence were greater when using sbTIV compared to eTIV (Figure 5a,c, respectively). A GLMM for adult longitudinal measurements showed no significant interaction terms (Table S7). However, the gap from age at initial scan and the gap by sex interaction were significant when using eTIV, suggesting that some eTIV especially for female participants were under‐estimated and showed increased eTIV in adult longitudinal measurements (Figure 5b).

## 4 Discussion

In this study, we tested the differences in TIV estimation methods for large‐scale samples from multi‐site MRI study projects. Although eTIV has been widely used for the estimation of TIV in FreeSurfer, we replicated the relatively low reliability compared to sbTIV, as previously reported [Cerri et al. 2023; Ma et al. 2019; Malone et al. 2015; Nerland et al. 2022; Puonti et al. 2016]. Notably, the discrepancy between the eTIV and sbTIV was prominent in those with a small TIV. The low reliability came from the scan protocols, and higher resolution and multimodal preprocessing did not always provide higher reliability. These inconsistencies are sensitive when testing longitudinal trajectories by using images from different procedures.

Consistent with previous comparisons [Nerland et al. 2022], we demonstrated higher validity and reliability of sbTIV compared to eTIV. Moreover, using multi‐site study datasets acquired with various protocols, we found that the protocols except for 1.0 mm isotropic resolution may provide smaller consistency between eTIV and sbTIV. Although the HARP and CRHD protocols fit the multimodal HCP pipeline for 0.8 mm isotropic T1‐weighted and T2‐weighted images, the ICCs were not excellent and slightly improved compared to the legacy mode using only T1‐weighted images. eTIV in the SRPB anisotropic images was underestimated but overestimated in the HARP and CRHD (0.8 mm isotropic) images, and the legacy mode pipeline decreased eTIV; the discrepancy trend may come from the usage of T2‐weighted images. One reason for this discrepancy may be that eTIV in FreeSurfer relies on a linear transformation to the T1w template, which was implemented for the 1.0 mm isotropic protocol. The images were resized to this resolution for images with other resolutions, leading to systematic estimation errors when misregistration occurred (Figure S1).

Notably, the discrepancy was sometimes remarkable for images with small TIV. The results showed that the low accuracy of eTIV showed across procedures, and it was difficult to predict which images resulted in underestimated eTIV calculations. Indeed, low accuracy is prominent in trajectory analysis of repeated scans, including TS. As cross‐sectional investigations from multiple sites may mimic this concern, we should carefully examine previous studies using eTIV, particularly for children and female studies. Even when longitudinal preprocessing pipelines are available, their outputs should be interpreted with caution, particularly for images acquired using different procedures and for studies of child and adolescent development.

On the other hand, we replicated the high consistency of sbTIV in these repeated scans [Cerri et al. 2023; Puonti et al. 2016]. The reliability is crucial in longitudinal investigations, and the present findings showed a more reliable developmental trajectory in adolescence and more stable volumes in adults and TS across procedures. Especially, high consistency was found between T1‐weighted image only and multimodal procedures, which would be helpful for future multi‐site studies with a mixture of T1‐weighted image only and multimodal datasets.

Because T2‐weighted images have a high contrast between the cerebrospinal fluid and skull, the T2‐weighted‐based or multimodal TIV estimation methods should be standard. However, owing to the strength of a large sample size of T1‐weighted image datasets, it is still necessary for TIV estimation only from T1‐weighted images, especially in multi‐site clinical studies. In addition, future studies should consider bias field correction of T1‐weighted and T2‐weighted images, such as radiofrequency transmit (B1+) field effects [Glasser et al. 2022; Haast et al. 2018]. Recent high‐resolution multimodal images are associated with a lower signal‐to‐noise ratio compared to conventional resolution, which results in a greater bias in the segmentation.

In conclusion, we showed that sbTIV is recommended for multi‐site studies using various imaging protocols. This recommendation is sensitive when observing longitudinal trajectories using various procedures. Although future studies will use more reliable estimation methods using multimodal images and considering bias fields, T1‐weighted‐based TIV estimation is still important because previous multi‐site study projects were based on the aggregation of T1‐weighted images. We also need to pay attention to previous studies that used eTIV as a confounding factor for structural features in multi‐site studies.

## Conflicts of Interest

The authors declare no conflicts of interest.

## Supporting information


**Appendix S1:** Supplementary information.

## Data Availability

Dataset from SRPBS DecNef project is publicly available from the SRPBS Multidisorder and Traveling Subject MRI Dataset and please request via https://bicr.atr.jp/decnefpro/data/. TS dataset from BMB‐HBM project is publicly available at the request of https://mridata‐brainminds‐beyond.atr.jp/dataset/bmbts/. The main dataset from the BMB‐HBM project is now being prepared for public sharing. The dataset of Procedure V and AD has not yet been opened publicly, and please contact the corresponding authors for the request before the opening.
