# Supplementary material for: Estimation of the Intracranial Volume Is Crucial in Multi‐Site Studies: Reliability for Longitudinal Investigations and Traveling Subjects
Source: Hum Brain Mapp. 2025 Nov 5;46(16):e70405. doi: 10.1002/hbm.70405 (PMC12587433; doi:10.1002/hbm.70405)
Supplement: Supplementary file 1 — Appendix S1: Supplementary information. [file HBM-46-e70405-s001.docx]

**Estimation of the intracranial volume is crucial in multi-site studies: Reliability for longitudinal investigations and traveling subjects**

Shinsuke Koike, M.D., Ph.D.^1,2,3,*^; Norihide Maikusa, M.D., Ph.D.^1^; Lin Cai, Ph.D.^1,4^; Issei Ueda, M.D., Ph.D.^1^; Shuhei Shibukawa, Ph.D.^1,5^; Toshihiko Aso, M.D., Ph.D.^6^; Saori C. Tanaka, Ph.D.^4,7^, Takuya Hayashi^5,8^; the Japanese Strategic Research Program for the Promotion of Brain Science (SRPBS) DecNef study project group; Brain/MINDS Beyond Human Brain MRI (BMB-HBM) study project group

Supplementary Materials

Table of Contents

[**Supplementary Methods** 3](#_Toc210639537)

[**Computing environments** 3](#_Toc210639538)

[**Image preprocessing pipeline** 3](#_Toc210639539)

[**Legacy-mode analysis for SRPB** 4](#_Toc210639540)

[**Patterns of Structural MRI Processing** 4](#_Toc210639541)

[**Additional preprocessing for sbTIV** 5](#_Toc210639542)

[**Longitudinal preprocessing pipelines** 5](#_Toc210639543)

[**References** 6](#_Toc210639544)

[**Supplementary Figures** 7](#_Toc210639545)

[**Supplementary Figure S1. Example of eTIV misregistration.** 7](#_Toc210639546)

[**Supplementary Figure S2. The relationship between eTIV and sbTIV by scan procedure.** 10](#_Toc210639547)

[**Supplementary Figure S3. Relationship between demographic characteristics and deviation and absolute difference of eTIV.** 11](#_Toc210639548)

[**Supplementary Figure S4. The difference in TIV estimation between preprocessing methods.** 11](#_Toc210639549)

[**Supplementary Figure S5. eTIV and sbTIV from longitudinal processing pipelines.** 12](#_Toc210639550)

[**Supplementary Figure S6. Relationship between age, body height, and TIV estimations.** 13](#_Toc210639551)

[**Supplementary Tables** 14](#_Toc210639552)

[**Supplementary Table S1. General linear models for intraclass correlation coefficients between procedures.** 14](#_Toc210639553)

[**Supplementary Table S2. General linear mixed models for deviation of eTIV.** 15](#_Toc210639554)

[**Supplementary Table S3. General linear mixed models for deviation of eTIV for non-small eTIV subgroup.** 16](#_Toc210639555)

[**Supplementary Table S4. General linear mixed models for absolute error of eTIV.** 17](#_Toc210639556)

[**Supplementary Table S5. General linear mixed models for absolute error of eTIV for non-small eTIV subgroup.** 18](#_Toc210639557)

[**Supplementary Table S6. Generalized additive mixed models for eTIV and sbTIV in adolescent longitudinal measurements.** 19](#_Toc210639558)

[**Supplementary Table S7. Generalized additive mixed models for eTIV and sbTIV in adult longitudinal measurements.** 20](#_Toc210639559)

# **Supplementary Methods**

## **Computing environments**

For the HARP protocol, Procedure A to S, all neuroimaging analyses were conducted using a Brain Connectomics Imaging High Performance Computing (BCI-HPC) system (Node: 12, CPU cores: 484, CPU: Xeon Gold 4316, GPU: NVIDIA A30, Memory, 1TB/node). For the CRHD and SRPB protocols, Procedures T to AD, all neuroimaging analyses were performed in a standardized computational environment comprising five dedicated analysis servers (Precision T7920 Tower Workstation, Dell Technologies Inc., TX) running Ubuntu 18.04 LTS (Canonical Ltd., London, UK). Each server was equipped with a 36- or 48-core Intel Xeon processor (Intel Corporation, CA) and 256 or 512 GB ECC memory, and all maintained identical software installations and environmental configurations. We verified that the output metrics from preprocessed images were identical across servers when the same raw brain image and preprocessing methods were applied.

## **Image preprocessing pipeline**

The Human Connectome Project (HCP) Pipelines implement the Minimal Preprocessing Pipelines (MPPs), providing a standardized framework for neuroimaging data preprocessing [Glasser et al., 2013]. The original HCP Pipelines operate in “HCPStyleData” mode, which enforces strict acquisition and processing requirements to ensure high-quality and reproducible results. These requirements include mandatory high-resolution T1-weighted and T2-weighted structural images, adherence to specific imaging parameters, and compliance with HCP acquisition guidelines.

The processing pipeline was implemented using HCP Pipelines version 4.3, with scanner-specific parameter for gradient non-linearity distortion correction using a gradient coefficient file for each Siemens scanner. Gradient nonlinearity distortion correction was not performed for the data from non-Siemens scanner.

Software Versions:

- HCP Pipelines: v4.3 (https://github.com/Wahington-University/HCPpipelines)
- gradunwarp.py: v1.2.3
- FreeSurfer: v6.0.1 (https://surfer.nmr.mgh.harvard.edu/)
- FSL: v6.0.7.14 (<https://fsl.fmrib.ox.ac.uk/fsl/>)
- Workbench Command: v2.0.1 (https://www.humanconnectome.org/software/workbench-command)
- MATLAB Runtime: R2023a (https://jp.mathworks.com/products/compiler/matlab-runtime.html)

## **Legacy-mode analysis for SRPB**

The research community has expressed a growing need to process existing datasets that do not fully conform to the stringent HCP standards. In response, the development team introduced the “LegacyStyleData” mode as an extension of the original pipeline framework [Glasser and Essen, 2011]. This legacy mode increases flexibility while preserving the core preprocessing methodology, thereby enabling researchers to apply standardized HCP preprocessing to datasets acquired under different protocols or with varying quality constraints.

The LegacyStyleData mode incorporates several key accommodations: T2-weighted images are optional rather than mandatory (as implemented in *FreeSurfer/FreeSurferPipeline.sh*); support is provided for older acquisition types such as single-band and low-resolution data; and alternative registration tools, such as FLIRT, can be applied when appropriate for legacy datasets (*fMRIVolume/GenericfMRIVolumeProcessingPipeline.sh*). The implementation also includes robust safety mechanisms through the processing mode check library (*global/scripts/processingmodecheck.shlib*), which issues explicit warnings regarding potential quality implications when deviating from standard HCP requirements.

The Legacy Mode represents a significant advancement in the practical utility of the HCP preprocessing pipelines, enabling broader adoption across diverse research datasets while preserving the methodological rigor of the original framework. This development allows researchers to leverage standardized preprocessing approaches even when working with data that predate or deviate from current HCP acquisition standards.

## **Patterns of Structural MRI Processing**

To ensure consistency across subjects, we implemented automated detection of processing patterns for structural MRI data, based on the available image types:

- HCPStyle (T2W-Extant_SEF-Extant): Full HCP-style processing with T1w, T2w, and spin-echo field maps
- HCPStyle_T1w_T2w_only (T2W-Extant_SEF-Absent): T1w and T2w processing without spin-echo field map distortion correction
- LegacyStyle_OnlyT1 (T2W-Absent_SEF-Absent): T1w-only processing without additional corrections
- LegacyStyle_with_TopupOnly (T2W-Absent_SEF-Extant): T1w processing with spin-echo field map distortion correction

## **Additional preprocessing for sbTIV**

Segmentation-based TIV (sbTIV) was estimated using the *Samseg* command in FreeSurfer, which performs whole-brain segmentation and computes sbTIV as the sum of intracranial label volumes [Puonti et al., 2016]. Unlike eTIV, sbTIV is a direct voxel-based estimate that does not rely on affine scaling. sbTIV can be derived either from T1-weighted (T1w) images alone or from multimodal T1w and T2-weighted (T2w) inputs, with identical computation procedures. In “HCPStyleData” mode, both T1w and T2w preprocessed images output by *PreFreeSurferPipeline.sh* (*T1w_acpc_dc_restore.nii.gz* and *T2w_acpc_dc_restore.nii.gz*, respectively) were used as inputs, whereas in “LegacyStyleData” mode, only the preprocessed T1w image was used. Since the *Samseg* command is supported only in FreeSurfer v7.2 and later, sbTIV estimation was performed using FreeSurfer v7.4.1.

## **Longitudinal preprocessing pipelines**

To evaluate the effect of the longitudinal preprocessing pipelines implemented in FreeSurfer, we additionally performed image preprocessing on six TS images from one participant and four images from one adolescent participant scanned in 8 years using the same software version as in the cross-sectional preprocessing. In the FreeSurfer longitudinal pipeline [Reuter et al., 2012], eTIV is derived once from the subject-specific base image, which serves as a within-subject template. This estimation of eTIV employs the same atlas-scaling factor method as used in the cross-sectional analysis.

In longitudinal sbTIV, all time points for a subject are processed jointly within a shared generative model, which enforces temporal consistency across sessions [Cerri et al., 2023]. sbTIV at each time point is then derived as the sum of intracranial labels, analogous to cross-sectional *Samseg* but with improved longitudinal stability.

# **References**

Cerri S, Greve DN, Hoopes A, Lundell H, Siebner HR, Mühlau M, Van Leemput K (2023): An open-source tool for longitudinal whole-brain and white matter lesion segmentation. Neuroimage Clin 38:103354.

Glasser MF, Essen DCV (2011): Mapping Human Cortical Areas In Vivo Based on Myelin Content as Revealed by T1- and T2-Weighted MRI. J Neurosci 31:11597–11616.

Glasser MF, Sotiropoulos SN, Wilson JA, Coalson TS, Fischl B, Andersson JL, Xu J, Jbabdi S, Webster M, Polimeni JR, Van Essen DC, Jenkinson M, WU-Minn HCP Consortium (2013): The minimal preprocessing pipelines for the Human Connectome Project. Neuroimage 80:105–124.

Puonti O, Iglesias JE, Van Leemput K (2016): Fast and sequence-adaptive whole-brain segmentation using parametric Bayesian modeling. Neuroimage 143:235–249.

Reuter M, Schmansky NJ, Rosas HD, Fischl B (2012): Within-subject template estimation for unbiased longitudinal image analysis. NeuroImage 61:1402–1418.

# **Supplementary Figures**


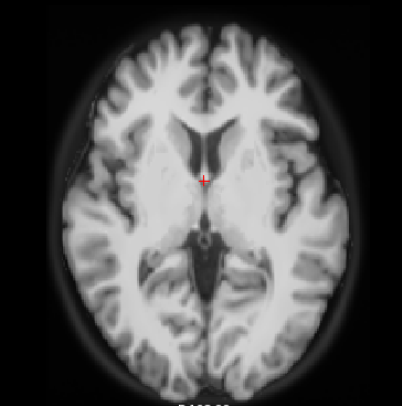

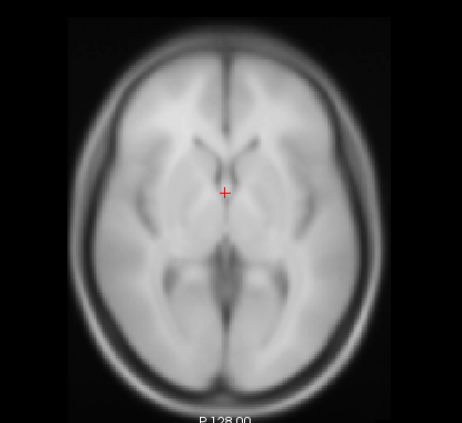


b

a


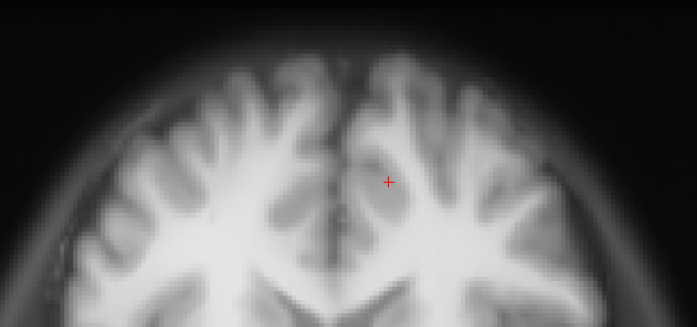


c

## **Supplementary Figure S1. Example of eTIV misregistration.**

Example of discrepancy between eTIV and sbTIV in Procedure AD. FreeSurfer software estimates eTIV by scaling the pre‑measured volume inside the skull of the MNI 305 template with the atlas‑scaling factor derived from the linear transformation to the native brain space (a). Therefore, its estimation depends on the linear transformation between the T1-weighted image and the atlas (b). When misregistration occurs between the image and atlas (c), eTIV calculates an incorrect value.

**
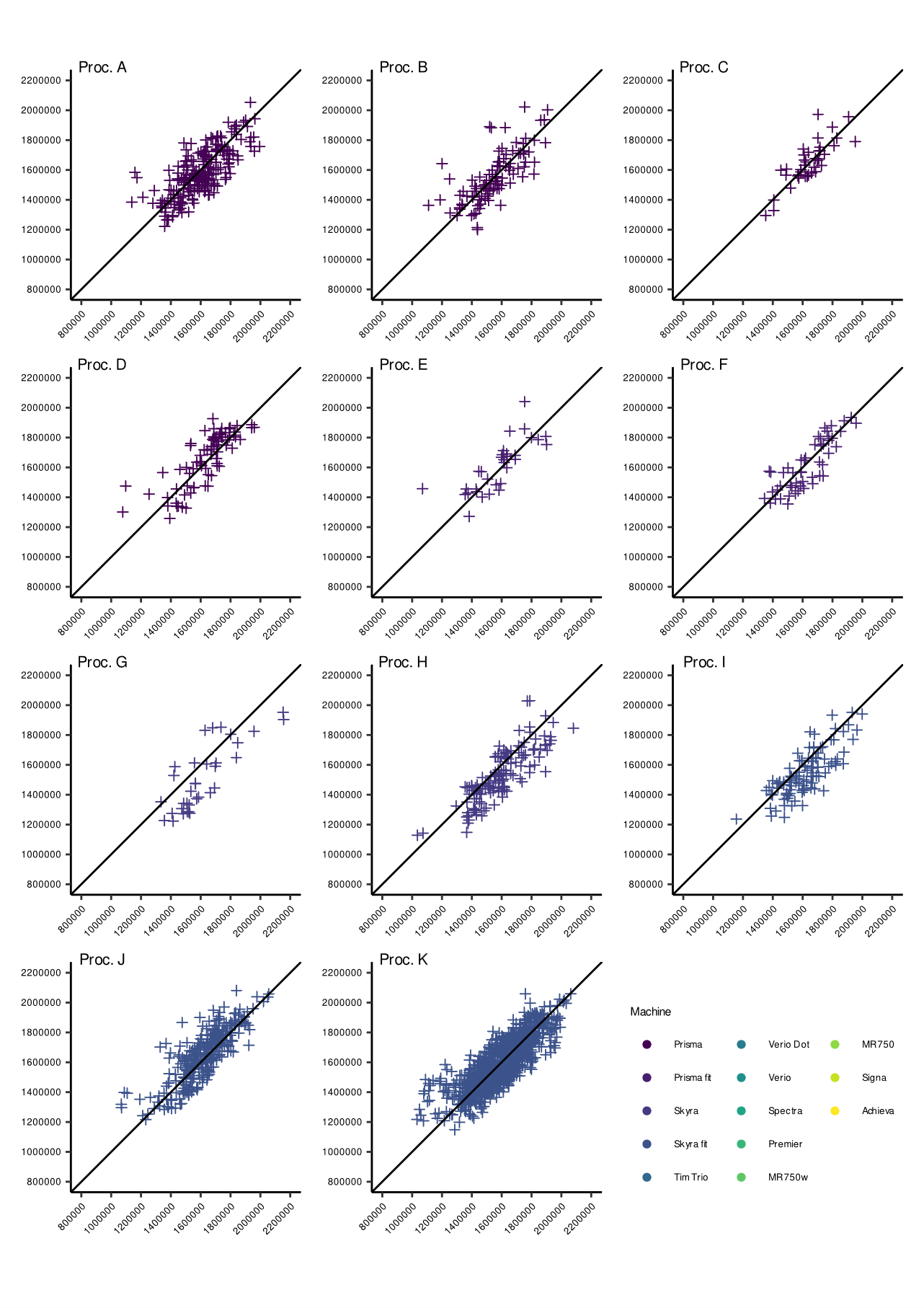
**

(Continue)


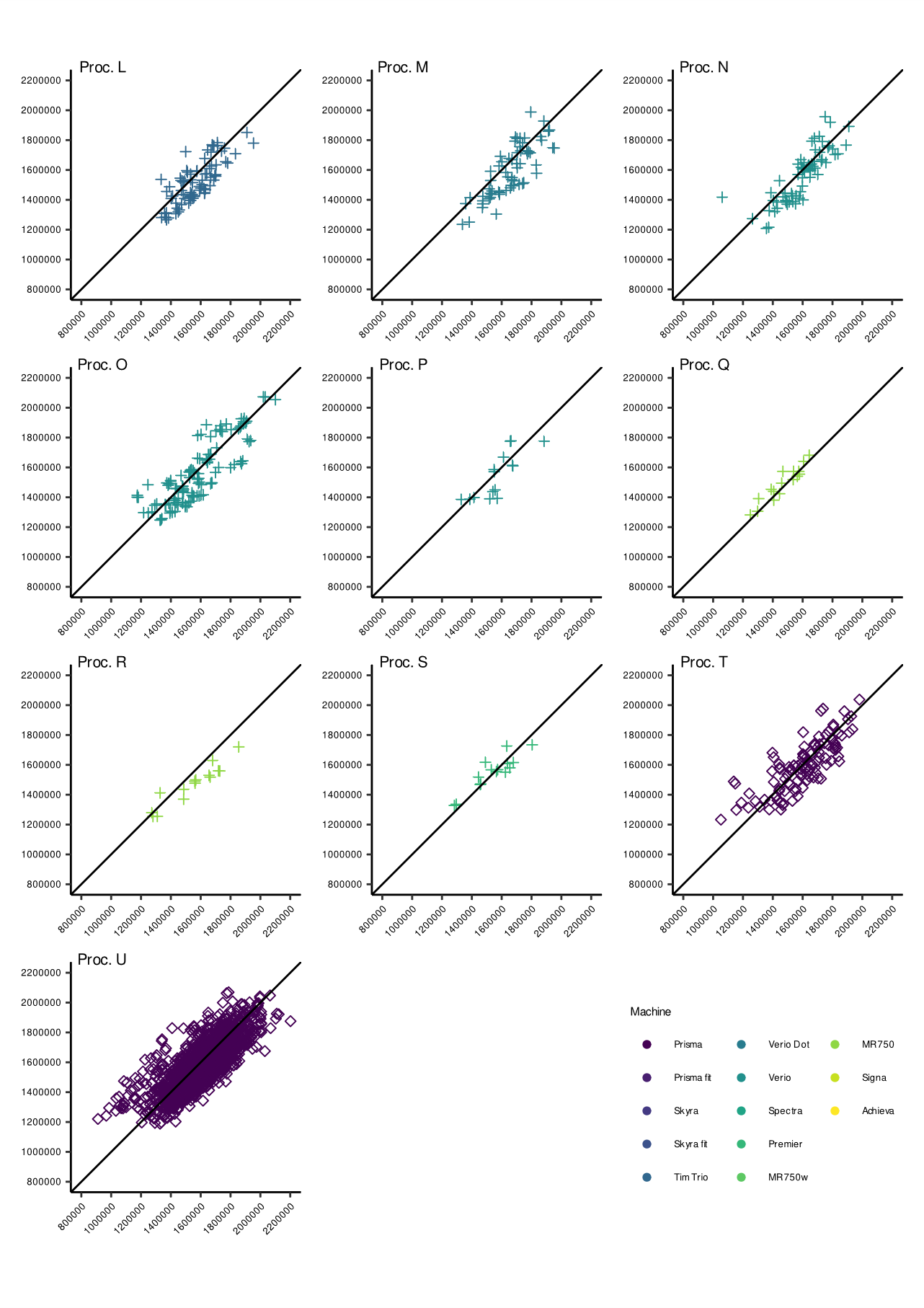


(Continue)


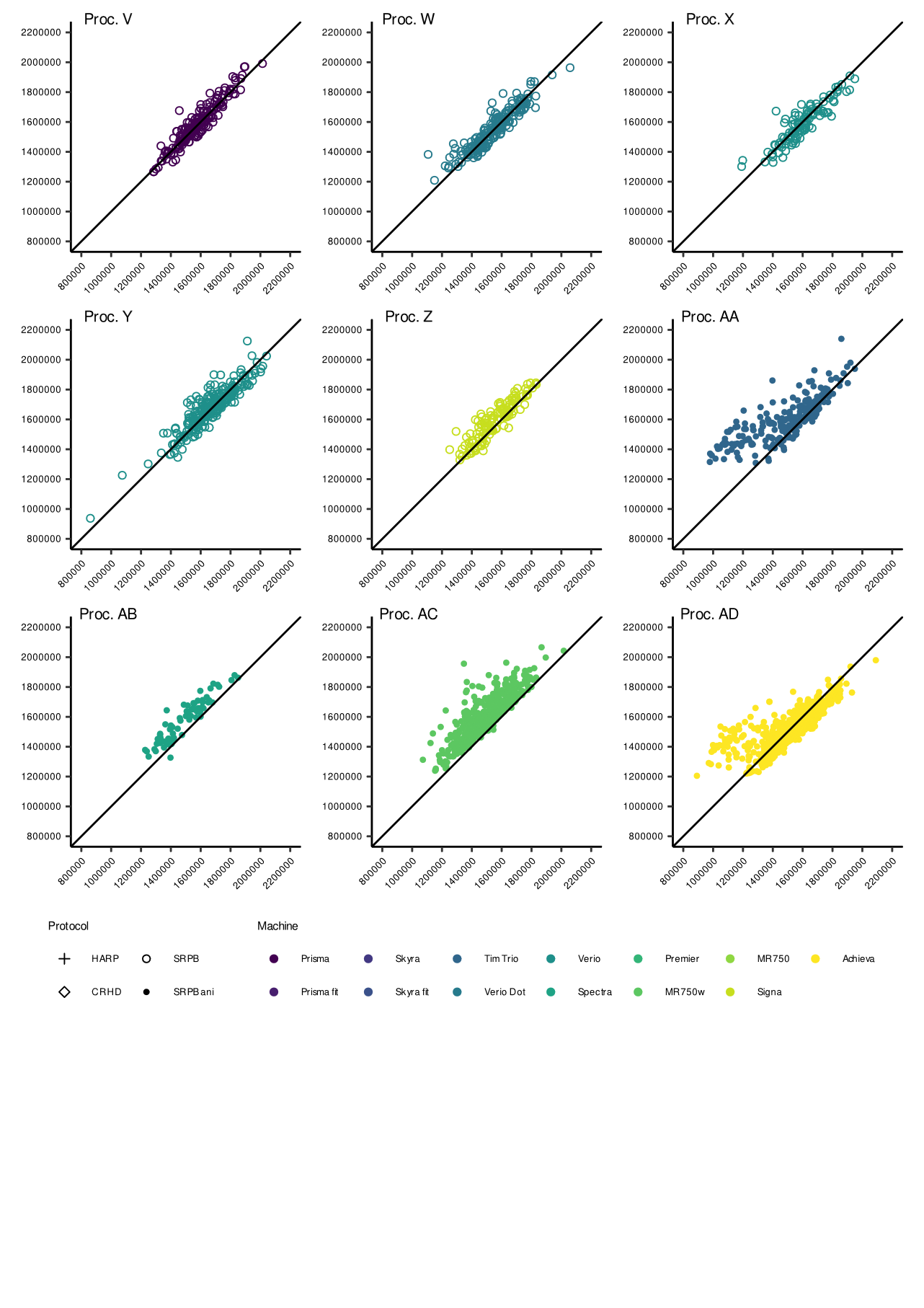


## **Supplementary Figure S2. The relationship between eTIV and sbTIV by scan procedure.**

The scan procedure tags correspond to the procedure codes in Tables 1 and 2. The X- and Y-axes show eTIV and sbTIV (mm^3^), respectively.


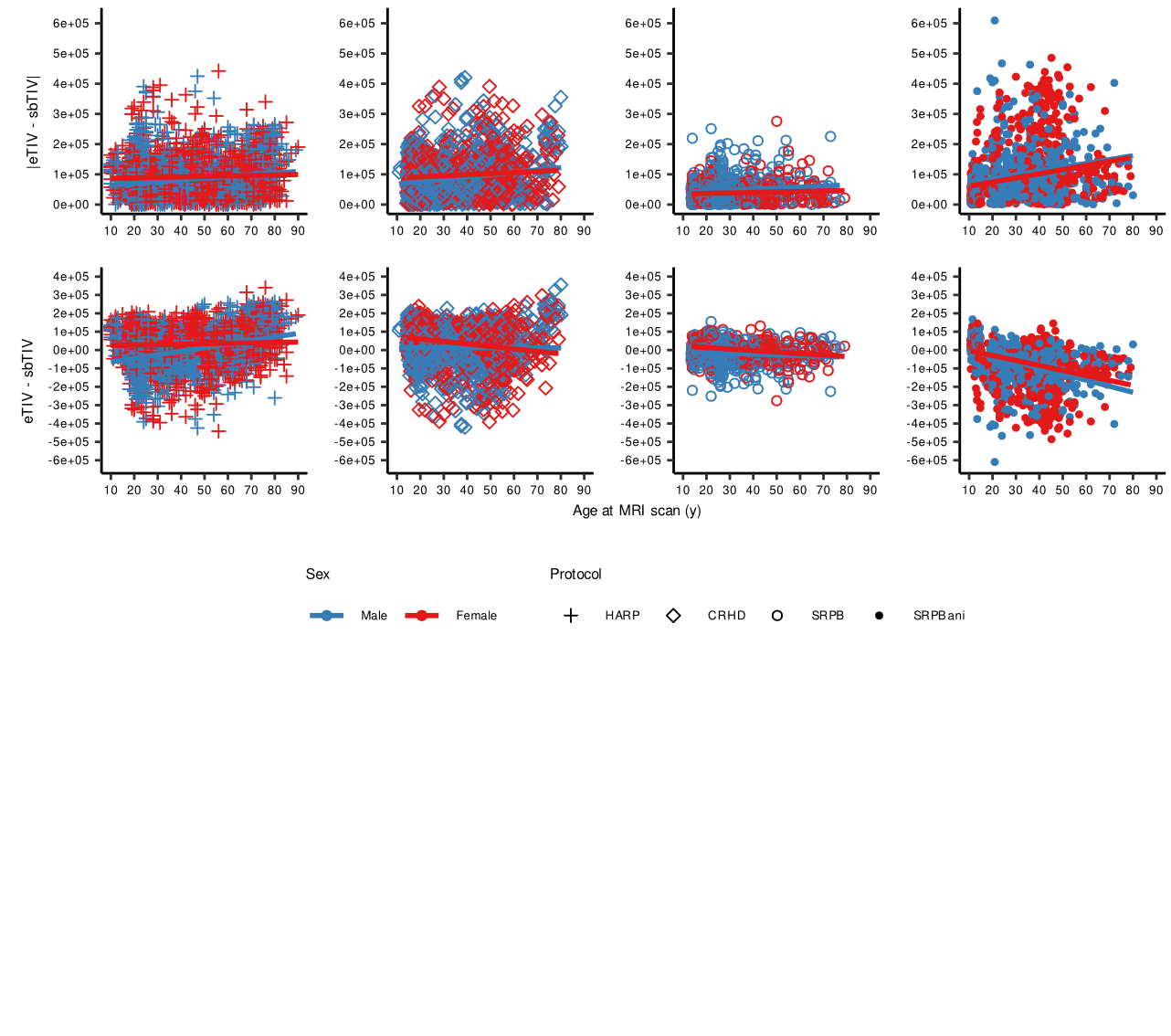


## **Supplementary Figure S3. Relationship between demographic characteristics and deviation and absolute difference of eTIV.**

**
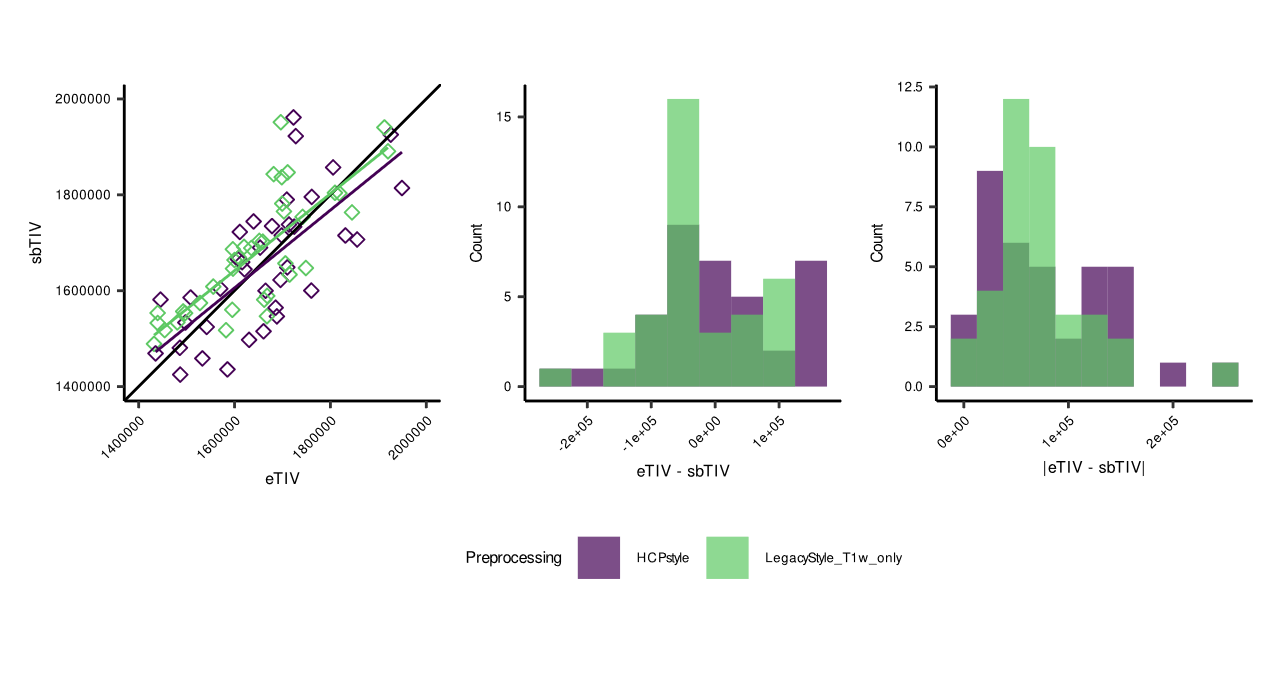
**

## **Supplementary Figure S4. The difference in TIV estimation between preprocessing methods.**

**
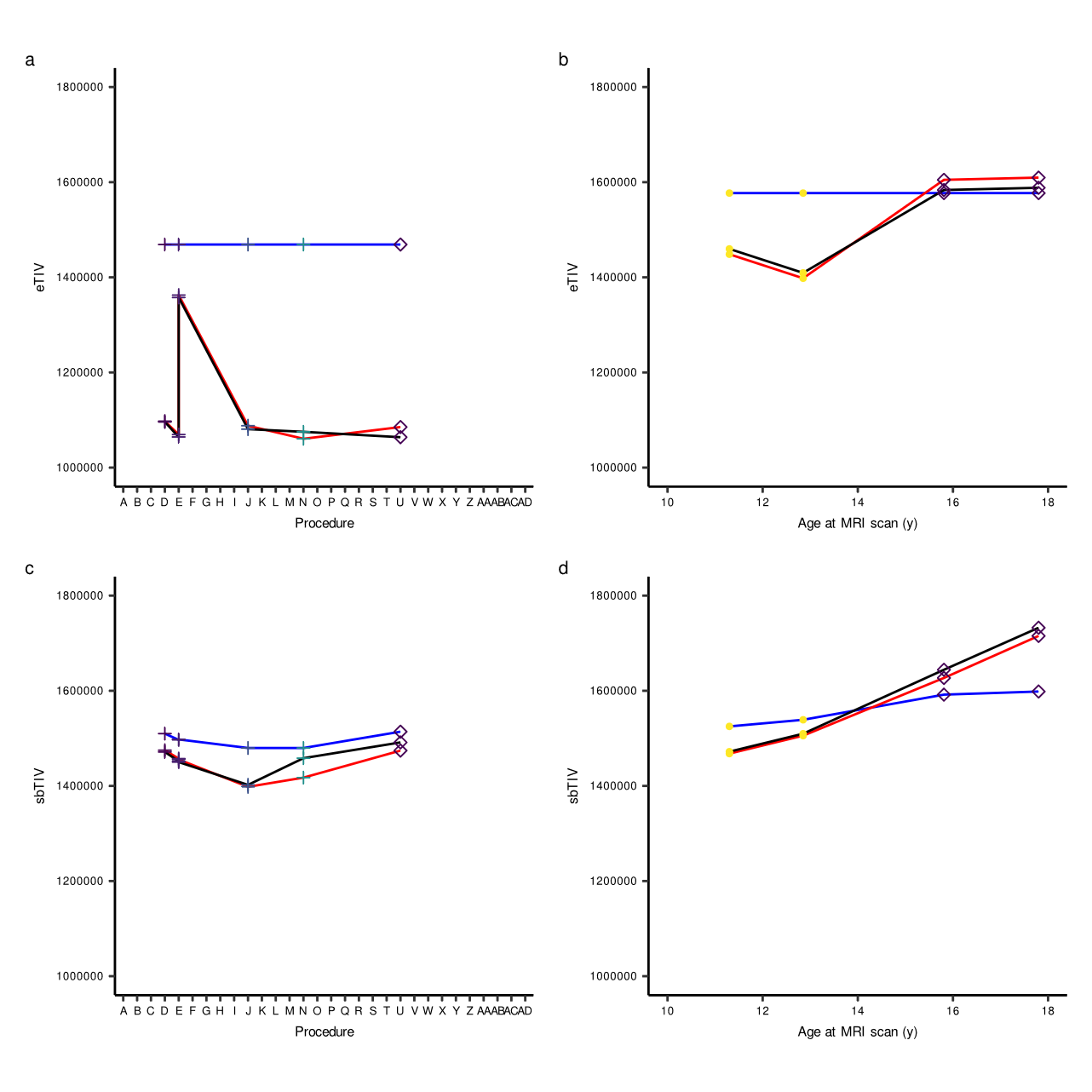
**

## **Supplementary Figure S5. eTIV and sbTIV from longitudinal processing pipelines.**

Raw (red) and harmonized (black) eTIV and sbTIV are illustrated for one TS participant with six images shown in **Figure 3** (a, c) and one adolescent participant with four images in 8 years (b, d). Blue lines show eTIV and sbTIV of longitudinal processing pipelines.


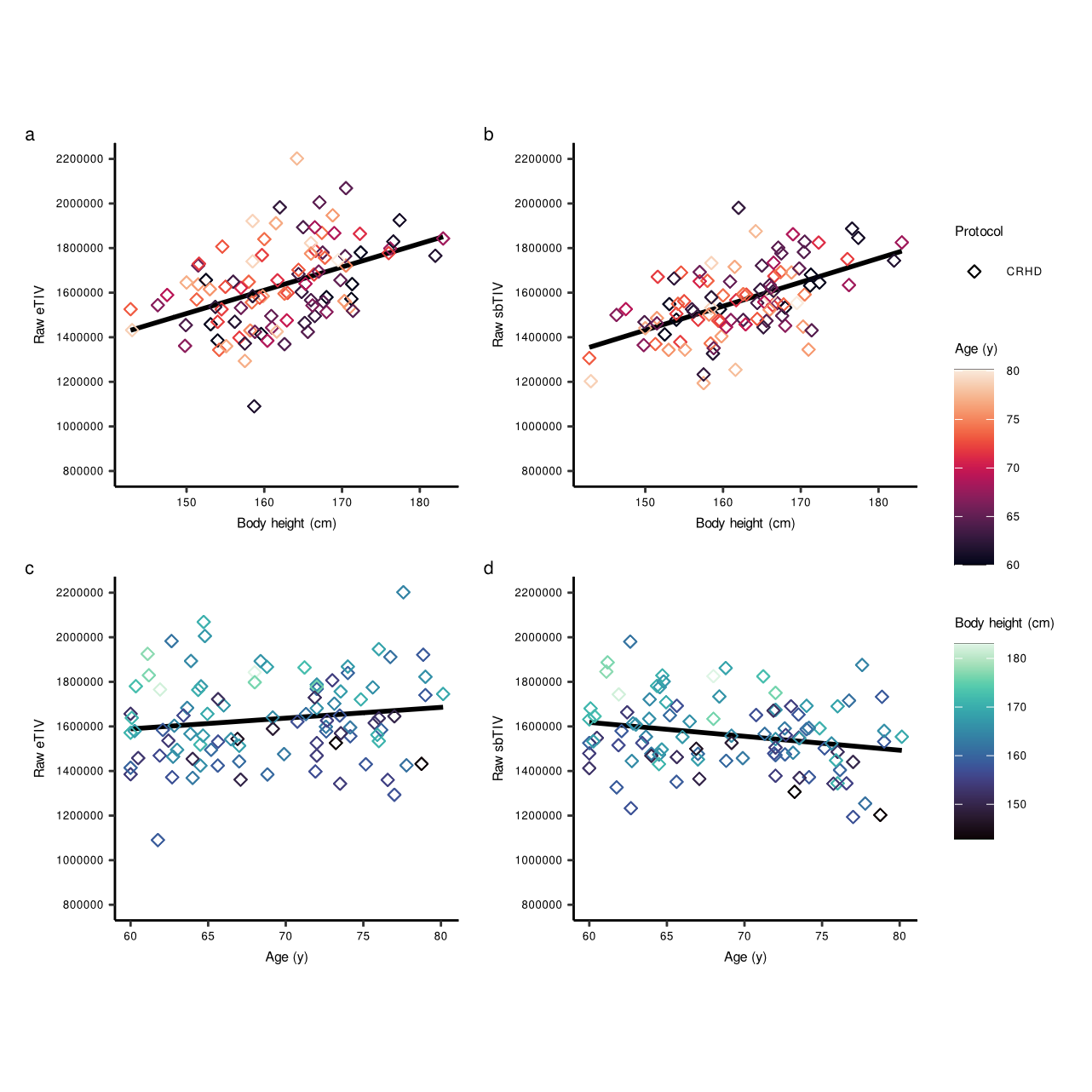


## **Supplementary Figure S6. Relationship between age, body height, and TIV estimations.**

# **Supplementary Tables**

## **Supplementary Table S1. General linear models for intraclass correlation coefficients between procedures.**

|  | **For all samples** | | | | **For non-small eTIV samples** | | | |
| --- | --- | --- | --- | --- | --- | --- | --- | --- |
|  | **Estimate** | **S.E.** | **t** | **p** | **Estimate** | **S.E.** | **t** | **p** |
| (Intercept) | **0.912** | **0.030** | **30.34** | **< 0.001** | **0.910** | **0.030** | **29.83** | **< 0.001** |
| Protocol |  |  |  |  |  |  |  |  |
| SRPB | (ref) |  |  |  |  |  |  |  |
| HARP | **-0.138** | **0.028** | **-4.89** | **< 0.001** | **-0.127** | **0.029** | **-4.46** | **0.001** |
| CRHD | **-0.133** | **0.044** | **-3.05** | **0.009** | **-0.131** | **0.044** | **-2.96** | **0.010** |
| SRPB ani | **-0.243** | **0.069** | **-3.52** | **0.003** | **-0.187** | **0.070** | **-2.68** | **0.018** |
| Machine |  |  |  |  |  |  |  |  |
| Prisma | (ref) |  |  |  |  |  |  |  |
| Prisma fit | 0.027 | 0.038 | 0.73 | 0.48 | 0.044 | 0.038 | 1.16 | 0.27 |
| Skyra | -0.026 | 0.038 | -0.69 | 0.50 | -0.046 | 0.038 | -1.20 | 0.25 |
| Skyra fit | 0.003 | 0.033 | 0.09 | 0.93 | -0.009 | 0.033 | -0.27 | 0.79 |
| Tim Trio | -0.046 | 0.049 | -0.93 | 0.37 | -0.053 | 0.050 | -1.07 | 0.30 |
| Verio Dot | -0.014 | 0.038 | -0.37 | 0.72 | -0.014 | 0.039 | -0.36 | 0.72 |
| Verio | 0.019 | 0.029 | 0.65 | 0.53 | 0.018 | 0.029 | 0.60 | 0.56 |
| Spectra | 0.105 | 0.080 | 1.32 | 0.21 | 0.053 | 0.081 | 0.65 | 0.53 |
| Premier | **0.113** | **0.049** | **2.31** | **0.037** | 0.106 | 0.050 | 2.12 | 0.052 |
| MR750w | -0.012 | 0.080 | -0.15 | 0.89 | -0.075 | 0.081 | -0.93 | 0.37 |
| MR750 | **0.097** | **0.038** | **2.58** | **0.022** | **0.090** | **0.038** | **2.35** | **0.034** |
| Signa | -0.045 | 0.054 | -0.85 | 0.41 | -0.043 | 0.054 | -0.78 | 0.45 |
| Achieva | 0.073 | 0.080 | 0.92 | 0.37 | 0.044 | 0.038 | 1.16 | 0.27 |

Bold shows significant (p < .05).

## **Supplementary Table S2. General linear mixed models for deviation of eTIV.**

|  | **Crude** | | | | | | **Mutually adjusted** | | | | | |
| --- | --- | --- | --- | --- | --- | --- | --- | --- | --- | --- | --- | --- |
|  | | **B** | **S.E.** | **df** | **t** | **p** | | **B** | **S.E.** | **df** | **t** | **p** |
| (Intercept) | | NA |  |  |  |  | | **-55776** | **19342** | **13.6** | **-2.88** | **0.012** |
| Protocol | |  |  |  |  |  | |  |  |  |  |  |
| SRPB | | (ref) |  |  |  |  | |  |  |  |  |  |
| HARP | | **38887** | **16821** | **24.0** | **2.31** | **0.030** | | **44533** | **17844** | **12.8** | **2.50** | **0.027** |
| CRHD | | 34984 | 27278 | 21.8 | 1.28 | 0.21 | | 56052 | 26746 | 11.3 | 2.10 | 0.059 |
| SRPB ani | | **-61830** | **21915** | **22.0** | **-2.82** | **0.010** | | -87305 | 42902 | 11.9 | -2.03 | 0.065 |
| Machine | |  |  |  |  |  | |  |  |  |  |  |
| Prisma | | (ref) |  |  |  |  | |  |  |  |  |  |
| Prisma fit | | -2000 | 30905 | 17.1 | -0.06 | 0.95 | | -4433 | 23796 | 12.7 | -0.19 | 0.86 |
| Skyra | | **73974** | **31497** | **18.2** | **2.35** | **0.030** | | **74170** | **24626** | **14.1** | **3.01** | **0.009** |
| Skyra fit | | 14499 | 26244 | 16.2 | 0.55 | 0.59 | | 12338 | 20479 | 11.8 | 0.60 | 0.56 |
| Tim Trio | | -33712 | 30549 | 16.3 | -1.10 | 0.29 | | 28879 | 30758 | 12.3 | 0.94 | 0.37 |
| Verio Dot | | 25224 | 30614 | 16.4 | 0.82 | 0.42 | | 46013 | 23822 | 12.0 | 1.93 | 0.077 |
| Verio | | 17097 | 22632 | 17.2 | 0.76 | 0.46 | | 33844 | 18296 | 13.0 | 1.85 | 0.087 |
| Spectra | | **-87594** | **41423** | **17.4** | **-2.11** | **0.049** | | 41557 | 50011 | 12.3 | 0.83 | 0.42 |
| Premier | | -39893 | 44143 | 22.4 | -0.90 | 0.38 | | -43997 | 34743 | 19.8 | -1.27 | 0.22 |
| MR750w | | **-117276** | **40319** | **15.7** | **-2.91** | **0.010** | | 10714 | 49143 | 11.4 | 0.22 | 0.83 |
| MR750 | | -491 | 34045 | 25.0 | -0.01 | 0.99 | | -4812 | 27614 | 22.6 | -0.17 | 0.86 |
| Signa | | -39333 | 40891 | 16.6 | -0.96 | 0.35 | | 1466 | 33451 | 12.0 | 0.04 | 0.97 |
| Achieva | | -18215 | 40127 | 15.4 | -0.45 | 0.66 | | 106878 | 49040 | 11.3 | 2.18 | 0.051 |
| Demographic characteristics | | | | | | | | | | | | |
| Age | | -164 | 95 | 4953.3 | -1.73 | 0.080 | | **-205** | **95** | **4461.4** | **-2.17** | **0.030** |
| Female | | **15981** | **3027** | **4496.4** | **5.28** | **< 0.001** | | **16483** | **3033** | **4496.3** | **5.43** | **< 0.001** |

Bold shows significant (p < .05). The deviation of eTIV from sbTIV (i.e., eTIV – sbTIV) was used as the dependent variable in the model.

## **Supplementary Table S3. General linear mixed models for deviation of eTIV for non-small eTIV subgroup.**

|  | **Crude** | | | | | | **Mutually adjusted** | | | | | |
| --- | --- | --- | --- | --- | --- | --- | --- | --- | --- | --- | --- | --- |
|  | | **B** | **S.E.** | **df** | **t** | **p** | | **B** | **S.E.** | **df** | **t** | **p** |
| (Intercept) | | NA |  |  |  |  | | **-75771** | **18473** | **13.6** | **-4.10** | **0.001** |
| Protocol | |  |  |  |  |  | |  |  |  |  |  |
| SRPB | | (ref) |  |  |  |  | |  |  |  |  |  |
| HARP | | **38214** | **17368** | **24.3** | **2.20** | **0.038** | | **42488** | **17065** | **12.9** | **2.49** | **0.027** |
| CRHD | | 40083 | 28324 | 22.5 | 1.42 | 0.17 | | **58511** | **25666** | **11.6** | **2.28** | **0.042** |
| SRPB ani | | **-49412** | **22745** | **22.7** | **-2.17** | **0.041** | | -50921 | 41156 | 12.2 | -1.24 | 0.24 |
| Machine | |  |  |  |  |  | |  |  |  |  |  |
| Prisma | | (ref) |  |  |  |  | |  |  |  |  |  |
| Prisma fit | | -7451 | 28143 | 16.9 | -0.26 | 0.79 | | -7899 | 22762 | 12.8 | -0.35 | 0.73 |
| Skyra | | **69031** | **28684** | **18.1** | **2.41** | **0.027** | | **67857** | **23477** | **14.1** | **2.89** | **0.012** |
| Skyra fit | | 11714 | 23908 | 16.1 | 0.49 | 0.63 | | 11487 | 19631 | 12.0 | 0.59 | 0.57 |
| Tim Trio | | -20358 | 27846 | 16.2 | -0.73 | 0.48 | | 26471 | 29435 | 12.4 | 0.90 | 0.39 |
| Verio Dot | | 22244 | 27878 | 16.3 | 0.80 | 0.44 | | 42008 | 22809 | 12.2 | 1.84 | 0.09 |
| Verio | | 15258 | 20612 | 17.1 | 0.74 | 0.47 | | 34773 | 17486 | 13.1 | 1.99 | 0.07 |
| Spectra | | **-90301** | **37716** | **17.3** | **-2.39** | **0.028** | | 1986 | 47908 | 12.4 | 0.04 | 0.97 |
| Premier | | -44467 | 40115 | 22.0 | -1.11 | 0.28 | | -46159 | 32802 | 19.0 | -1.41 | 0.18 |
| MR750w | | **-120861** | **36731** | **15.6** | **-3.29** | **0.005** | | -29292 | 47188 | 11.7 | -0.62 | 0.55 |
| MR750 | | -5063 | 30933 | 24.6 | -0.16 | 0.87 | | -7048 | 25995 | 21.4 | -0.27 | 0.79 |
| Signa | | -43309 | 37241 | 16.4 | -1.16 | 0.26 | | -2890 | 32036 | 12.1 | -0.09 | 0.93 |
| Achieva | | -4374 | 36556 | 15.3 | -0.12 | 0.91 | | 84101 | 47104 | 11.6 | 1.79 | 0.10 |
| Demographic characteristics | | | | | | | | | | | | |
| Age | | -16 | 87 | 4866.2 | -0.18 | 0.86 | | -89 | 86 | 4475.6 | -1.03 | 0.30 |
| Female | | **31214** | **2770** | **4371.6** | **11.27** | **< 0.001** | | **31377** | **2776** | **4372.9** | **11.30** | **< 0.001** |

Bold shows significant (p < .05). The deviation of eTIV from sbTIV (i.e., eTIV – sbTIV) was used as the dependent variable in the model.

## **Supplementary Table S4. General linear mixed models for absolute error of eTIV.**

|  | **Crude** | | | | | | **Mutually adjusted** | | | | | |
| --- | --- | --- | --- | --- | --- | --- | --- | --- | --- | --- | --- | --- |
|  | | **B** | **S.E.** | **df** | **t** | **p** | | **B** | **S.E.** | **df** | **t** | **p** |
| (Intercept) | | NA |  |  |  |  | | **24903** | **7372** | **7.9** | **3.38** | **0.010** |
| Protocol | |  |  |  |  |  | |  |  |  |  |  |
| SRPB | | (ref) |  |  |  |  | |  |  |  |  |  |
| HARP | | **43888** | **7549** | **20.2** | **5.81** | **< 0.001** | | **39243** | **6566** | **6.3** | **5.98** | **0.001** |
| CRHD | | **46535** | **11845** | **16.5** | **3.93** | **0.001** | | **44902** | **8987** | **3.9** | **5.00** | **0.008** |
| SRPB ani | | **53448** | **9552** | **17.0** | **5.60** | **< 0.001** | | **61960** | **15177** | **5.3** | **4.08** | **0.008** |
| Machine | |  |  |  |  |  | |  |  |  |  |  |
| Prisma | | (ref) |  |  |  |  | |  |  |  |  |  |
| Prisma fit | | -5287 | 18035 | 17.3 | -0.29 | 0.77 | | -6961 | 9058 | 7.7 | -0.77 | 0.47 |
| Skyra | | 38040 | 18346 | 18.2 | 2.07 | 0.053 | | 14879 | 9401 | 7.2 | 1.58 | 0.16 |
| Skyra fit | | 2856 | 15081 | 15.4 | 0.19 | 0.85 | | -4627 | 7086 | 4.6 | -0.65 | 0.54 |
| Tim Trio | | 21904 | 17609 | 15.7 | 1.24 | 0.23 | | 8370 | 11227 | 6.2 | 0.75 | 0.48 |
| Verio Dot | | -15917 | 17679 | 16.0 | -0.90 | 0.38 | | -6126 | 8528 | 5.5 | -0.72 | 0.50 |
| Verio | | -16054 | 13159 | 17.0 | -1.22 | 0.24 | | -298 | 6809 | 6.4 | -0.04 | 0.97 |
| Spectra | | 8065 | 24074 | 17.4 | 0.34 | 0.74 | | -21576 | 18116 | 6.1 | -1.19 | 0.28 |
| Premier | | -16189 | 26882 | 26.8 | -0.60 | 0.55 | | -14469 | 16636 | 27.8 | -0.87 | 0.39 |
| MR750w | | 32561 | 23062 | 14.6 | 1.41 | 0.18 | | 5373 | 16807 | 4.5 | 0.32 | 0.76 |
| MR750 | | -590 | 20915 | 30.9 | -0.03 | 0.98 | | 816 | 13620 | 33.7 | 0.06 | 0.95 |
| Signa | | -30791 | 23571 | 16.0 | -1.31 | 0.21 | | 3377 | 11808 | 5.4 | 0.29 | 0.79 |
| Achieva | | -10489 | 22916 | 14.3 | -0.46 | 0.65 | | -34850 | 16680 | 4.4 | -2.09 | 0.10 |
| Demographic characteristics | | | | | | | | | | | | |
| Age | | **468** | **65** | **4082.9** | **7.25** | **< 0.001** | | **441** | **64** | **946.7** | **6.94** | **< 0.001** |
| Female | | **6891** | **2080** | **4333.3** | **3.31** | **0.001** | | **5496** | **2072** | **4286.1** | **2.65** | **0.008** |

Bold shows significant (p < .05). The absolute error of eTIV from sbTIV (i.e., |eTIV – sbTIV|) was used as the dependent variable in the model.

## **Supplementary Table S5. General linear mixed models for absolute error of eTIV for non-small eTIV subgroup.**

|  | **Crude** | | | | | | **Mutually adjusted** | | | | | |
| --- | --- | --- | --- | --- | --- | --- | --- | --- | --- | --- | --- | --- |
|  | | **B** | **S.E.** | **df** | **t** | **p** | | **B** | **S.E.** | **df** | **t** | **p** |
| (Intercept) | | NA |  |  |  |  | | **39471** | **8000** | **9.1** | **4.93** | **0.001** |
| Protocol | |  |  |  |  |  | |  |  |  |  |  |
| SRPB | | (ref) |  |  |  |  | |  |  |  |  |  |
| HARP | | **44212** | **8227** | **21.2** | **5.37** | **< 0.001** | | **40532** | **7257** | **7.9** | **5.59** | **0.001** |
| CRHD | | **43183** | **13159** | **18.5** | **3.28** | **0.004** | | **43567** | **10402** | **5.9** | **4.19** | **0.006** |
| SRPB ani | | **41638** | **10588** | **18.7** | **3.93** | **0.001** | | 29371 | 17132 | 7.0 | 1.71 | 0.13 |
| Machine | |  |  |  |  |  | |  |  |  |  |  |
| Prisma | | (ref) |  |  |  |  | |  |  |  |  |  |
| Prisma fit | | -1502 | 17624 | 17.1 | -0.09 | 0.93 | | -4311 | 9878 | 8.8 | -0.44 | 0.67 |
| Skyra | | **42212** | **17860** | **17.9** | **2.36** | **0.030** | | 22807 | 10228 | 9.1 | 2.23 | 0.053 |
| Skyra fit | | 4899 | 14806 | 15.5 | 0.33 | 0.75 | | -3690 | 8067 | 6.5 | -0.46 | 0.66 |
| Tim Trio | | 7898 | 17288 | 15.9 | 0.46 | 0.65 | | 10092 | 12444 | 7.6 | 0.81 | 0.44 |
| Verio Dot | | -13813 | 17325 | 16.0 | -0.80 | 0.44 | | -2660 | 9544 | 7.2 | -0.28 | 0.79 |
| Verio | | -14455 | 12860 | 17.0 | -1.12 | 0.28 | | -1342 | 7514 | 8.1 | -0.18 | 0.86 |
| Spectra | | 9702 | 23490 | 17.1 | 0.41 | 0.68 | | 13273 | 20157 | 7.6 | 0.66 | 0.53 |
| Premier | | -13711 | 25766 | 24.7 | -0.53 | 0.60 | | -14305 | 16534 | 23.0 | -0.87 | 0.40 |
| MR750w | | 34269 | 22696 | 14.9 | 1.51 | 0.15 | | 39992 | 19269 | 6.3 | 2.08 | 0.081 |
| MR750 | | 1895 | 19934 | 27.8 | 0.10 | 0.92 | | 1168 | 13386 | 27.6 | 0.09 | 0.93 |
| Signa | | -28279 | 23092 | 16.0 | -1.22 | 0.24 | | 6341 | 13264 | 6.9 | 0.48 | 0.65 |
| Achieva | | -23303 | 22584 | 14.6 | -1.03 | 0.32 | | -14291 | 19191 | 6.2 | -0.74 | 0.48 |
| Demographic characteristics | | | | | | | | | | | | |
| Age | | **347** | **58** | **4191.9** | **6.03** | **< 0.001** | | **349** | **57** | **1980.9** | **6.10** | **< 0.001** |
| Female | | **-3874** | **1855** | **4213.0** | **-2.09** | **0.037** | | **-4846** | **1851** | **4208.4** | **-2.62** | **0.009** |

Bold shows significant (p < .05). The absolute error of eTIV from sbTIV (i.e., |eTIV – sbTIV|) was used as the dependent variable in the model.

## **Supplementary Table S6. Generalized additive mixed models for eTIV and sbTIV in adolescent longitudinal measurements.**

|  | **All** | | | | | **eTIV** | | | | | **sbTIV** | | | | | |
| --- | --- | --- | --- | --- | --- | --- | --- | --- | --- | --- | --- | --- | --- | --- | --- | --- |
|  | **B** | **S.E.** | **df** | **t** | **p** | **B** | **S.E.** | **df** | **t** | **p** | **B** | **S.E.** | **df** | **t** | **p** |  |
| (Intercept) | **1476557** | **31476** | **2324** | **46.9** | **<.001** | **1480883** | **33998** | **1225** | **43.6** | **<.001** | **1262885** | **25971** | **1235** | **48.6** | **<.001** |  |
| Age (y) | **22722** | **1771** | **2096** | **12.8** | **<.001** | **22466** | **1912** | **864.1** | **11.8** | **<.001** | **35810** | **1381** | **852.9** | **25.9** | **<.001** |  |
| Sex |  |  |  |  |  |  |  |  |  |  |  |  |  |  |  |  |
| Male | (ref) |  |  |  |  |  |  |  |  |  |  |  |  |  |  |  |
| Female | -28747 | 20229 | 2320 | -1.4 | 0.16 | -37111 | 21826 | 1226 | -1.7 | 0.089 | **94609** | **16680** | **1234.3** | **5.7** | **<.001** |  |
| Estimation |  |  |  |  |  |  |  |  |  |  |  |  |  |  |  |  |
| eTIV | (ref) |  |  |  |  | NA |  |  |  |  | NA |  |  |  |  |  |
| sbTIV | **-208712** | **36172** | **2064** | **-5.8** | **<.001** | NA |  |  |  |  | NA |  |  |  |  |  |
| Age x sex | **-8115** | **1135** | **2095** | **-7.2** | **<.001** | **-7589** | **1223** | **863.3** | **-6.2** | **<.001** | **-17386** | **883** | **852.4** | **-19.7** | **<.001** |  |
| Age x Est. | **12782** | **2337** | **2064** | **5.5** | **<.001** | NA |  |  |  |  | NA |  |  |  |  |  |
| Sex x Est. | **114862** | **23269** | **2064** | **4.9** | **<.001** | NA |  |  |  |  | NA |  |  |  |  |  |
| Age x Sex x Est. | **-8728** | **1500** | **2064** | **-5.8** | **<.001** | NA |  |  |  |  | NA |  |  |  |  |  |

Bold shows significant (p < .05).

## **Supplementary Table S7. Generalized additive mixed models for eTIV and sbTIV in adult longitudinal measurements.**

|  | **All** | | | | | **eTIV** | | | | | **sbTIV** | | | | |
| --- | --- | --- | --- | --- | --- | --- | --- | --- | --- | --- | --- | --- | --- | --- | --- |
|  | **B** | **S.E.** | **df** | **t** | **p** | **B** | **S.E.** | **df** | **t** | **p** | **B** | **S.E.** | **df** | **t** | **p** |
| (Intercept) | **1903567** | **21385** | **521.1** | **89.0** | **<.001** | **1913519** | **24639** | **427.2** | **77.7** | **<.001** | **1909455** | **19417** | **426.8** | **98.3** | **<.001** |
| Gap (y) | -6691 | 7279 | 1492 | -0.9 | .36 | **-14467** | **4775** | **527.4** | **-3.0** | **.003** | 2023 | 3587 | 526.8 | 0.56 | .57 |
| Sex |  |  |  |  |  |  |  |  |  |  |  |  |  |  |  |
| Male | (ref) |  |  |  |  |  |  |  |  |  |  |  |  |  |  |
| Female | **-223005** | **13339** | **521.7** | **-16.7** | **<.001** | **-232932** | **15367** | **427.6** | **-15.2** | **<.001** | **-221342** | **12110** | **427.2** | **-18.3** | **<.001** |
| Estimation |  |  |  |  |  |  |  |  |  |  |  |  |  |  |  |
| eTIV | (ref) |  |  |  |  | NA |  |  |  |  | NA |  |  |  |  |
| sbTIV | 15908 | 12173 | 1437.0 | 1.3 | .19 | NA |  |  |  |  | NA |  |  |  |  |
| Gap x sex | 6718 | 3815 | 1492.1 | 1.8 | .078 | **13413** | **2504** | **527.5** | **5.4** | **<.001** | -1334 | 1881 | 526.9 | -0.7 | .48 |
| Gap x Est. | -214 | 9285 | 1437.0 | -0.02 | .98 | NA |  |  |  |  | NA |  |  |  |  |
| Sex x Est. | -7764 | 7581 | 1437.0 | -1.0 | .31 | NA |  |  |  |  | NA |  |  |  |  |
| Gap x Sex x Est. | -1063 | 4865 | 1437.0 | -0.22 | .83 | NA |  |  |  |  | NA |  |  |  |  |

Bold shows significant (p < .05).
